# *Vibrio natriegens* as a pET-Compatible Expression Host Complementary to *Escherichia coli*

**DOI:** 10.3389/fmicb.2021.627181

**Published:** 2021-02-19

**Authors:** Jiaqi Xu, Feng Dong, Meixian Wu, Rongsheng Tao, Junjie Yang, Mianbin Wu, Yu Jiang, Sheng Yang, Lirong Yang

**Affiliations:** ^1^Institute of Bioengineering, College of Chemical and Biological Engineering, Zhejiang University, Hangzhou, China; ^2^Huzhou Center of Industrial Biotechnology, Shanghai Institutes for Biological Sciences, Huzhou, China; ^3^Key Laboratory of Synthetic Biology, CAS Center for Excellence in Molecular Plant Sciences, Chinese Academy of Sciences, Shanghai, China; ^4^Huzhou Yisheng Biotechnology Co., Ltd., Huzhou, China; ^5^Shanghai Taoyusheng Biotechnology Co., Ltd., Shanghai, China

**Keywords:** *Vibrio natriegens*, recombinant protein, pET expression system, fermentation optimization, synthetic biology

## Abstract

Efficient and novel recombinant protein expression systems can further reduce the production cost of enzymes. *Vibrio natriegens* is the fastest growing free-living bacterium with a doubling time of less than 10 min, which makes it highly attractive as a protein expression host. Here, 196 pET plasmids with different genes of interest (GOIs) were electroporated into the *V. natriegens* strain VnDX, which carries an integrated T7 RNA polymerase expression cassette. As a result, 65 and 75% of the tested GOIs obtained soluble expression in *V. natriegens* and *Escherichia coli*, respectively, 20 GOIs of which showed better expression in the former. Furthermore, we have adapted a consensus “what to try first” protocol for *V. natriegens* based on Terrific Broth medium. Six sampled GOIs encoding biocatalysts enzymes thus achieved 50–128% higher catalytic efficiency under the optimized expression conditions. Our study demonstrated *V. natriegens* as a pET-compatible expression host with a spectrum of highly expressed GOIs distinct from *E. coli* and an easy-to-use consensus protocol, solving the problem that some GOIs cannot be expressed well in *E. coli*.

## Introduction

The *Escherichia coli* pET expression system remains one of the most popular systems for recombinant protein production (Shilling et al., 2020). Numerous strategies have been proposed to improve recombinant protein production, including codon optimization of target proteins (Burgess-Brown et al., 2008; Menzella, 2011; Chung and Lee, 2012), screening and construction of a superior host cell (Choi et al., 2015; Liu et al., 2015; Hayat et al., 2018), addition of fusion tags (Esposito and Chatterjee, 2006; Young et al., 2012; Hayat et al., 2018), optimization of expression elements (Gustafsson et al., 2012; Eichmann et al., 2019), or the development of high-density fermentation processes (Yee and Blanch, 1992; Zhang et al., 2000; Thongekkaew et al., 2008). Although *E. coli* is highly amenable to genetic modification and has a clear genetic background, quite many genes of interest (GOIs) were not well-expressed in *E. coli*. Furthermore, contamination of fermentation processes with other microorganisms or phages is a common problem that requires an urgent solution (Samson et al., 2013; Ou et al., 2020). Developing alternative hosts that possess a distinct spectrum of highly expressed GOIs complementary to *E. coli* and compatible with the pET expression system is an attractive way to solve this problem (Calero and Nikel, 2019).

*Vibrio natriegens*, a Gram-negative, non-pathogenic marine bacterium (Payne, 1958), is considered the fastest growing free-living bacterium, with a doubling time between 7 and 10 min under optimal conditions (Eagon, 1962; Weinstock et al., 2016; Lee et al., 2019). Estimates for the number of ribosomes in *V. natriegens* in the exponential phase suggest around 115,000 per cell, while *E. coli* is estimated to have only 70,000–90,000 (Des Soye et al., 2018), which partly explains its greater biomass synthesis rate and stronger protein expression ability (Zhu et al., 2020). Therefore, *V. natriegens* as the host for GOI expression is allowed to own higher productivity, saving time and energy costs in industrial production (Hoffart et al., 2017).

With the development of genetic tools or methods for *V. natriegens* since 2016 (Weinstock et al., 2016; Dalia et al., 2017), studies investigated multiple aspects of this organism, including fast growth and metabolism (Long et al., 2017; Lee et al., 2019; Pfeifer et al., 2019), expression of recombinant protein (Levarská et al., 2018; Becker et al., 2019; Eichmann et al., 2019; Tschirhart et al., 2019), establishment of cell-free protein synthesis system (CFPS) (Des Soye et al., 2018; Failmezger et al., 2018; Wiegand et al., 2018), and synthesis of chemicals (Liu, 2002; Fernandez-Llamosas et al., 2017; Ellis et al., 2019; Wang et al., 2019; Erian et al., 2020). The T7 RNA polymerase cassette containing the gene encoding T7 RNA polymerase under the control of an isopropyl β-D-1-thiogalactopyranoside (IPTG) inducible promoter *lacUV5* was necessary for pET protein expression system. The *dns* gene in *V. natriegens* encoding Dns nuclease reduces the integrity of plasmid DNA (Weinstock et al., 2016). Therefore, they developed the commercial *V. natriegens* Vmax strain by integrating the T7 RNA polymerase cassette at *dns* locus and successfully induced green fluorescent protein (GFP) expression using plasmid pET-28a-GFP (Weinstock et al., 2016). It has also been reported that isotopically labeled proteins FK506-binding protein (FKBP) and enhanced yellow fluorescent protein (EYFP) (Becker et al., 2019), as well as recombinant human growth hormone (hGT), yeast alcohol dehydrogenase (ADH), and archaeal catalase-peroxidase (AfKatG) (Kormanova et al., 2020), were successfully produced using the pET expression system, among which FKBP, EYFP, and AfKatG showed better soluble expression than in *E. coli* BL21(DE3). Besides, the membrane protein Mrp from the secondary transport system of *Vibrio cholerae* was also functionally expressed in *V. natriegens* (Schleicher et al., 2018). However, despite its high application potential as a novel recombinant protein expression host, the recombinant GOI production capacity and highly expressed spectrum of *V. natriegens* have not been comprehensively evaluated.

Here, we electroporated each of 196 recombinant pET plasmids expressing commonly used biocatalysts enzymes (Jiang et al., 2016) into the non-pathogenic and fastest-growing *V. natriegens* ATCC14048 with an integrated T7 RNA polymerase cassette in advance. Analysis of the soluble expression of these GOIs in both *V. natriegens* VnDX and *E. coli* BL21(DE3) showed that the spectrum of highly expressed GOIs in *V. natriegens* was complementary to that of *E. coli*. Furthermore, we adapted a consensus “what to try first” fermentation protocol for *V. natriegens* based on Terrific Broth (TB) Salt (TBv2) medium. These findings confirm that *V. natriegens* has great potential as a novel cell factory for recombinant protein production, which is compatible with the pET system and complementary to *E. coli*.

## Results

### Evaluation of the Soluble Expression of 196 Genes of Interest and Analysis of the Spectrum of Highly Expressed Genes of Interest of *V. natriegens*

The T7 RNA polymerase cassette and spectinomycin resistance cassette used for positive screening were integrated into the *dns* locus in the genome of *V. natriegens* ATCC14048, resulting in a modified *V. natriegens* strain VnDX (Supplementary Figure 1). The pET-24a-GFP plasmid transformants of VnDX cultured under the same conditions as that of Vmax (Weinstock et al., 2016) resulted in similar GFP fluorescence (Supplementary Figure 1), indicating that the VnDX can be considered equivalent for recombinant protein production using the pET system to Vmax.

**FIGURE 1:**
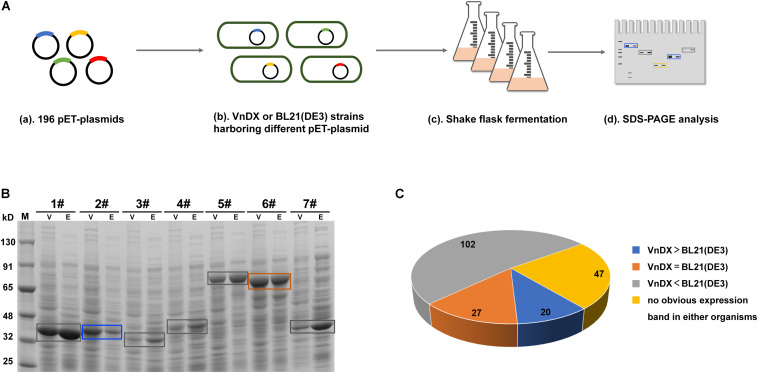
Evaluation of the soluble expression of 196 genes of interest (GOIs) in *Escherichia coli* and *Vibrio natriegens*. **(A)** Flowchart about shake-flask fermentation of 196 GOIs and soluble expression comparison between *E. coli* BL21(DE3) and *V. natriegens* VnDX. **(B)** Compare the soluble expression of the same GOI in *E. coli* and *V. natriegens* through sodium dodecyl sulfate polyacrylamide gel electrophoresis (SDS-PAGE). Here, 0.15 OD_600_ (10-well gel) or 0.075 OD_600_ (15-well gel) prepared crude protein samples were loaded to the corresponding protein gel wells. V, *V. natriegens* VnDX; E, *E. coli* BL21(DE3); Blue box, GOI possessing higher expression in VnDX than BL21(DE3); Orange box, GOI possessing the same expression in VnDX and BL21(DE3); Gray box, GOI possessing higher expression in BL21(DE3) than VnDX. 1#: Alanine racemase from *Bacillus subtilis* 168; 2#: N-acetyl amino acid racemase from *Alcaligenes* sp.; 3#: N-acetyl amino acid racemase from *Deinococcus radiodurans* NCHU1003; 4#: N-acetyl amino acid racemase from *Amycolatopsis* sp. TS-1-60; 5#: Maltooligosaccharide trehalose synthetase from *Arthrobacter* sp. Q36; 6#: Maltooligosaccharide trehalose synthetase from *Arthrobacterium* S34; 7#: D-carbamoylase (K34E) from *Burkholderia pickettii*. **(C)** Pie chart about the soluble expression results of 196 GOIs. VnDX > BL21(DE3): GOI possessing higher expression in VnDX than BL21(DE3); VnDX = BL21(DE3): GOI possessing the same expression in VnDX and BL21(DE3); VnDX < BL21(DE3): GOI possessing higher expression in BL21(DE3) than VnDX.

In a previous study, we established an enzyme library (Supplementary Table 1) containing 196 GOIs, including many commonly used biocatalyst enzymes (Jiang et al., 2016), the *Saccharomyces cerevisiae* sodium–potassium pump Trk1 (Ko and Gaber, 1991), and plant-derived isoprenoid synthase and its mutants (Yang et al., 2016), covering seven families of enzymes from bacteria, fungi, and plants. The pET expression plasmids encoding the GOIs were electroporated into *V. natriegens* VnDX and *E. coli* BL21(DE3) to compare the two expression systems. The optimal expression condition for *E. coli* BL21(DE3) (Sun et al., 2020) and the reported expression condition for *V. natriegens* (Weinstock et al., 2016) were used for flask fermentation to compare the soluble expression of each GOI in the two microbial hosts (Figure 1A). Finally, the quantitative amounts of the soluble protein per OD_600_ were compared between *V. natriegens* and *E. coli* (Figure 1B and Supplementary Figure 2).

**FIGURE 2:**
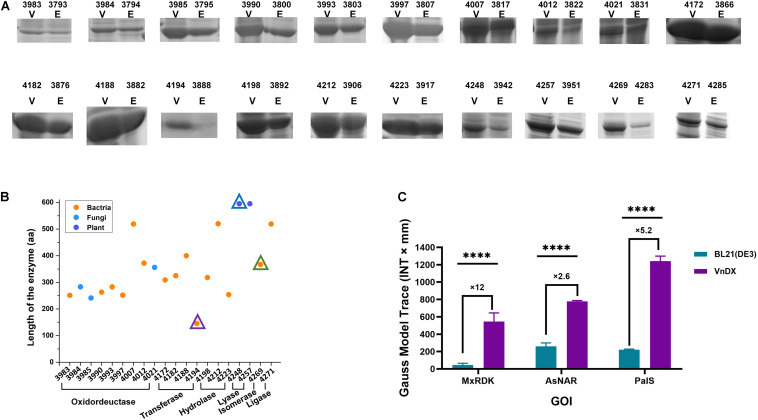
Analysis of the spectrum of highly expressed genes of interest (GOIs) in *V. natriegens* VnDX. **(A)** Sodium dodecyl sulfate polyacrylamide gel electrophoresis (SDS-PAGE) comparison of 20 highly expressed GOIs. Here, 0.15 OD_600_ (10-well gel) or 0.075 OD_600_ (15-well gel) prepared crude protein samples were loaded to the corresponding protein gel wells. V, *V. natriegens* VnDX; E, *E. coli* BL21(DE3). Numbers above the SDS-PAGE lanes represent strain numbers of 20 highly expressed GOIs in both hosts. The detailed information of these GOIs was marked with blue asterisk in Supplementary Table 3. **(B)** Classification, sources, and protein length analysis of 20 GOIs with high expression in *V. natriegens*. Numbers of horizontal axis represents *V. natriegens* strain numbers of 20 highly expressed GOIs. The GOI indicated by the three triangles corresponds to three enzymes in **(C)**. **(C)** Quantity comparison of three highly expressed GOIs with poor expression in *E. coli*. MxRDK: Ribonucleoside diphosphate kinase from *M. xanthus*, CIBT3888 and CIBT4194; AsNAR: N-acetyl amino acid racemase from *Alcaligenes* sp. CIBT3942 and CIBT4248; PaIS: Isoprene synthase (K308R, C533W) from *Populus alba* (codon Optimized for *E. coli*) CIBT4283 and CIBT4269. Error bars represent the SD of *n* = 3 technical replicates. Quantitative analysis was calculated by software Quantity One 1-D; GraphPad Prism software was used to analyze the significance by *t-*test, ****P < 0.0001.

We evaluated the expression results of 196 GOIs (Figure 1C) and found that the expression of 102 GOIs in *V. natriegens* was not as good as in *E. coli*, while no significant overexpression bands were observed for 47 GOIs in either organism. However, we also found that 27 GOIs were expressed equally in both hosts, and 20 GOIs were expressed at higher levels than in *E. coli* (Figure 2A), unexpectedly, although the fermentation conditions of *V. natriegens* were not optimized.

The 20 highly expressed GOIs of *V. natriegens* (Figure 2B) encompass 6 families of enzymes except for translocase (Supplementary Figure 3A), indicating that *V. natriegens* can express practically any type of enzyme. Analyzing the source of all GOIs revealed that the spectrum of highly expressed proteins of *V. natriegens* was derived from various species, including bacteria such as *E. coli* and *Lactobacillus kefiri*, fungi such as *Candida magnoliae*, and even plants (*Populus alba*) (Supplementary Figure 3B). The length of the 196 GOIs ranged from 100 to 1,000 amino acids (aa), while highly expressed GOIs in *V. natriegens* were all within 100–600 aa, suggesting that *V. natriegens* may have an advantage in the production of enzymes with small molecular weight (Supplementary Figure 3C).

Moreover, ribonucleoside diphosphate kinase from *Myxococcus xanthus*, N-acetyl amino acid racemase from *Alcaligenes* sp. and isoprene synthase (K308, C533W) from *Populus alba* (codon optimized for *E. coli*), showed poor expression in *E. coli* BL21(DE3) according to sodium dodecyl sulfate polyacrylamide gel electrophoresis (SDS-PAGE), while having obvious soluble expression bands indicating excellent expression in *V. natriegens*. Further quantification revealed that the expression of N-acetyl amino acid racemase in *V. natriegens* was 2.6 times higher than that in *E. coli*, and that of isoprene synthase was 5.2 times higher. Ribonucleoside diphosphate kinase showed the most significant difference with 12 times higher expression in *V. natriegens* than in *E. coli* (Figure 2C). These three proteins are isomerase, lyase, and transferase, respectively. Moreover, they are derived from different species and have significantly different molecular weights. Thus, *V. natriegens* can be viewed as a complementary protein expression host to *E. coli*.

Our overall assessment indicates that the modified *V. natriegens* is compatible with the commonly used pET expression system and has a different spectrum of highly expressed GOIs from *E. coli*. Moreover, enzymes from all sources and all families can potentially be expressed using the pET system in *V. natriegens*. In addition, we also found that plant-derived enzymes were highly expressed in *V. natriegens*. Therefore, it can be used as a host for some GOIs that are difficult to express in *E. coli*, acting as a complementary expression host alongside *E. coli*.

### Optimizing the Culture Medium for Growth and Protein Production

Because its enzyme activity can be measured conveniently (Sun et al., 2020; Zhang et al., 2020), we chose glucose dehydrogenase (GDH) from *Bacillus subtilis* as a reporter enzyme to screen culture media for optimal expression in *V. natriegens*. TB is the most recommended medium for protein production in *E. coli* (Gräslund et al., 2008; Novagen). Aiming to produce a better protein expression medium than the medium based on Brain Heart Infusion (BHI) and Luria–Bertani (LB) (Weinstock et al., 2016; Kormanova et al., 2020), an additional 15 g/L NaCl was added to TB (Hoffart et al., 2017), resulting in the modified TBv2 medium.

**FIGURE 3:**
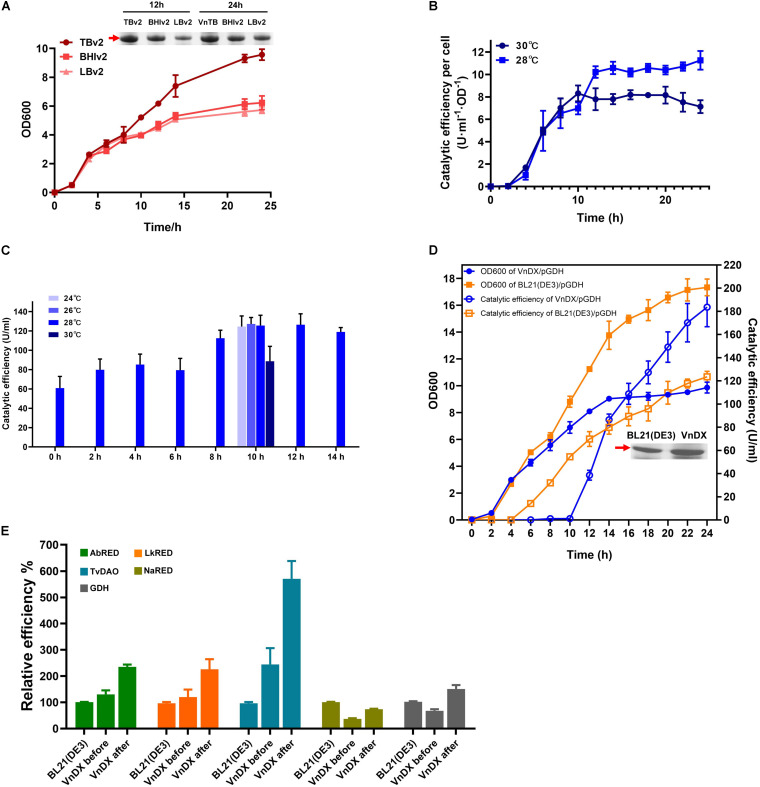
Optimization of “what to try first” protocol for *V. natriegens* and testing the general applicability of the consensus protocol. **(A)** Growth curve of VnDX/pGDH in TBv2, BHIv2, and LBv2 medium and sodium dodecyl sulfate polyacrylamide gel electrophoresis (SDS-PAGE) analysis of soluble glucose dehydrogenase (GDH) in 12 and 24 h. Here, 0.075 OD_600_ crude protein samples were loaded for SDS-PAGE. **(B)** The curve of GDH catalytic efficiency per OD_600_ [U/(ml⋅OD)] at 28 or 30°C. Induction occurred at 0 h. **(C)** Optimization of induction point and temperature of VnDX/pGDH. **(D)** Compare growth and catalytic efficiency of GDH between VnDX and BL21(DE3) under consensus “what to try first” fermentation conditions. **(E)** Five genes of interest (GOIs) were tested for the general applicability of the “what to try first” protocol. AbRED, carbonyl reductase from *Acinetobacter baylyi* CIBT3993; LkRED, carbonyl reductase from *Lactobacillus kefiri* CIBT3995; TvDAO, D-amino acid oxidase from *Trigonopsis variabilis* CIBT4021; NaRED, carbonyl reductase from *Novosphingobium aromaticivorans* DSM 12444 CIBT3990; GDH, glucose dehydrogenase from *Bacillus subtilis*. BL21(DE3), the optimal fermentation condition for *E. coli* BL21(DE3); VnDX before, *V. natriegens* fermentation condition before optimization; VnDX after, the fermentation condition using our improved “what to try first” protocol. Error bars represent the SD of *n* = 3 technical replicates.

Growth of VnDX/pGDH was evaluated in BHIv2, TBv2, and LBv2 culture medium. There was no significant difference between the media during 0–8 h. However, the growth in TBv2 was much better than BHIv2 or LBv2 after 8 h. The final cell density in TBv2 was 50% higher than in BHIv2 and that in LBv2 was 16% lower than that in BHIv2. According to SDS-PAGE analysis, the GDH expression after 24-h fermentation in TBv2 was 59% higher than in BHIv2, while in LBv2, it was 48% lower than in BHIv2 (Figure 3A).

To further verify whether TBv2 has generally better for recombinant protein production in *V. natriegens*, four enzymes were selected from the list of GOIs with poorer expression than *E. coli*: simvast acyltransferase from *Aspergillus terreus*, D-amino acid transaminase from *Silicibacter promeroyi* DSS-3, haine racemase from *Agrobacterium tumefaciens* str. C58, and maltooligosaccharide trehalose hydrolase from *Arthrobacterium 34*. In all cases, TBv2 was still the best medium for growth and protein expression (Supplementary Figure 4). Therefore, TBv2 can be generally recommended for protein production using the pET system in *V. natriegens*.

### A Consensus “What to Try First” Protocol for Improving Catalytic Efficiency in *V. natriegens*

Kormanova et al. (2020) optimized the fermentation conditions for the expression of hGH, ADH, and AfkatG in *V. natriegens*, respectively, but obtained inconsistent results. Therefore, a more general protocol for *V. natriegens* was needed, giving researchers the first-to-try choice for protein production in this novel host. We aimed to adapt a consensus “what to try first” protocol in TBv2 for *V. natriegens* based on our collective analysis of the expression of over 200 GOIs in *E. coli* BL21(DE3) (Gräslund et al., 2008; Jiang et al., 2016; Rongsheng et al., 2016).

When the induction temperature of VnDX/pGDH was decreased from 30 to 28°C, which was a “first-to-try” temperature for *E. coli* (Tao et al., 2014), the catalytic efficiency of GDH (U/ml) increased by 35% (Supplementary Figure 5B). This increase was not due to an increase in cell density (Supplementary Figure 5A) but resulted from an increase of GDH yield per OD_600_ by 57% (Figure 3B). We observed a turning point in catalytic efficiency per OD_600_ at nearly 10–12 h, so it was considered that adding an inducer at the 10th to the 12th hour might further increase GDH yield.

Therefore, we explored the optimal point in time to add the inducer after the seed culture was transferred to the shake flask (we called “induction point”). Eight trials were conducted, each being a multiple of two from 0 to 14 h, the highest titer of 130 U/ml was reached when it was induced at 10 h (Figure 3C). We then tested two other induction temperatures in the range of 24–30°C. The reduction of the temperature from 30 to 28°C did improve the GDH catalytic efficiency, but there was no obvious benefit from a further reduction of induction temperature (Figure 3C). Considering that the continuous reduction of temperature in industrial applications will greatly increase energy consumption, 28°C was selected as the “what to try first” induction temperature for *V. natriegens*.

The cell growth and catalytic efficiency of *V. natriegens* VnDX/pGDH and *E. coli* BL21(DE3)/pGDH were compared under the optimized “what to try first” conditions. Although the cell density of *V. natriegens* was 35% lower than that of *E. coli* at 24 h, the GDH yield of *V. natriegens* had overtaken that of *E. coli* after 4 h of induction. The catalytic efficiency reached 185 U/ml at the end of fermentation, which was 60% higher than that in *E. coli* (Figure 3D). Moreover, the yield per OD_600_ was 2.44 times higher than that of *E. coli* (Supplementary Figure 6), indicating that the higher catalytic efficiency of *V. natriegens* was due to superior expression of soluble GDH per unit of biomass. The possible explanation for this phenomenon was that the recombinant expression of toxic GDH in *V. natriegens* caused a greater burden on cells, so its growth rate was slightly lower than that under optimal conditions.

Our results indicate that choosing an appropriate induction temperature and induction point could balance cell growth and recombinant protein expression more effectively, which greatly enhanced the final catalytic efficiency of GDH.

### Testing the General Applicability of the Consensus “What to Try First” Protocol

The GDH catalytic efficiency increased by 123% in *V. natriegens* using the “what to try first” protocol (Figure 3E). To further confirm this consensus protocol, we also tested the expression of carbonyl reductase from *Acinetobacter baylyi* (AbRED), carbonyl reductase from *Lactobacillus kefiri* (LkRED), and D-amino acid oxidase from *Trigonopsis variabilis* (TvDAO). These were chosen for convenience, since their enzyme activity assays were the simplest among the 20 enzymes that showed better expression in *V. natriegens* than in *E. coli*. Additionally, we also tested carbonyl reductase from *Novosphingobium aromaticivorans* DSM 12444 (NaRED), which showed inferior expression in *V. natriegens*. Compared with the fermentation conditions reported in the literature, the enzyme efficiency increased by 82% for LkRED, 76% for AbRED, 128% for TvDAO, and 100% for NaRED. Furthermore, while the NaRED efficiency in *V. natriegens* was only 33% of that in *E. coli* before optimization, it increased to 60% under the optimized conditions (Figure 3E).

Thus, to adapt the *E. coli* consensus “what to try first” protocol based on TBv2 medium for *V. natriegens*, we changed the temperature to optimally grown temperature 30°C (Supplementary Figure 7) and induction point to the 10th hour at 28°C for *V. natriegens* in this study, and we proved that this modified protocol was effective for various GOIs.

## Discussion

This study compared the soluble expression of 196 GOI encoding enzymes of various families from various sources between *V. natriegens* VnDX and *E. coli* BL21(DE3). The results showed that the engineered *V. natriegens* strain VnDX is compatible with the pET system but had a distinct spectrum of highly expressed proteins compared with *E. coli*, indicating that it might be a valuable complementary expression system to *E. coli*. A consensus “what to try first” protocol for *V. natriegens* was obtained through fermentation optimization, which significantly improved the expression of GDH, TvDAO, NaRED, LkRED, and AbRED. Moreover, this protocol also enabled the soluble expression of other recombinant GOIs in *V. natriegens*, indicating its general applicability.

While comparison of the insoluble or total expression should give us an in-depth insight into the capacity of protein synthesis in both *E. coli* and *V. natriegens*, our limited resources were allocated to evaluation of the soluble protein output from more GOIs in *V. natriegens* rather than the total recombinant protein yield in both hosts. Nevertheless, the insoluble fractions of three GOIs in both organisms were run on SDS-PAGE and the amount of recombinant protein looked negligible (Supplementary Figure 8). The success rate of soluble expression was 75% in *E. coli*, while it reached 65% in *V. natriegens*. The soluble expression of 129 GOIs in *V. natriegens* was less than or equal to that of *E. coli*. There are three possible explanations for this phenomenon: (1) Almost all the GOIs were codon-optimized for *E. coli*. While GOIs codon-optimized for *V. natriegens* and *E. coli*, respectively, should avoid biases, considering that *E. coli* is the first-to-try chassis for protein expression, it will save time and cost in practice to transform pET plasmids that were not well-expressed in *E. coli* into *V. natriegens* directly. For the researchers who are dedicated to optimize their GOI expression in *V. natriegens* or believe *V. natriegens* is the better expression host at the beginning, they had better synthesize their GOIs with *V. natriegens* codon optimization. (2) The spectrum of highly expressed was different in *V. natriegens* and *E. coli*. Therefore, the two hosts are compatible. (3) *E. coli* BL21(DE3) produced proteins under optimized fermentation conditions, while *V. natriegens* used suboptimal conditions and our consensus “what to try first” protocol increased the yield for many GOIs. Therefore, further optimization should be considered when using *V. natriegens* as an industrial expression host in the future.

It is known that differences in media composition affect microbial recombinant protein production (Broedel et al., 2001; Terol et al., 2019). Furthermore, it was reported that the stability of expression plasmids could be improved by using complex nitrogen sources (Matsui et al., 1990). Here, we reported TBv2 for the first time, a medium recommended for *V. natriegens* fermentation. We believe that several factors contribute to the satisfactory results of protein production in TBv2: its carbon source was more abundant than those of LBv2 and BHIv2; a higher concentration of yeast extract increased background expression of lacUV5 promoter (Doran et al., 1990; Gomes and Mergulhao, 2017), which may improve the protein expression of the T7 system; TBv2 contains glycerin (Clomburg and Gonzalez, 2013; Terol et al., 2019), and supplementing complex media with glycerol improves protein production (Terol et al., 2019).

Maximization of protein production and minimization of costs are major aims in the production of recombinant proteins, which requires a balance between cell growth and the expression of recombinant proteins (Gomes and Mergulhao, 2017). It has been reported that de-coupling cell growth from protein production in *E. coli* could increase the yield of various proteins (Lemmerer et al., 2019; Stargardt et al., 2020). We found that the accumulation of biomass at the optimal temperature in the early fermentation stage and induction of protein expression in the later stage were also effective strategies to improve the yield of recombinant proteins in *V. natriegens*. These results showed that appropriate separation between the growth of *V. natriegens* and protein production could better balance the resources in the cell and achieve the goal of maximizing protein production.

Because different proteins had different optimal expression conditions, there can be no “right” answer *a priori* (Gräslund et al., 2008), which is the same in *V. natriegens* (Kormanova et al., 2020). In order to facilitate the decision-making, we compared with the general fermentation method for *E. coli* established in our laboratory (Jiang et al., 2016), proposed a consensus “what to try first” protocol for *V. natriegens* and verified the effectiveness of this protocol in the expression of multiple GOIs. This protocol provides a reference for researchers who are starting out with protein expression in *V. natriegens*. When they are uncertain about appropriate fermentation conditions for a protein, they can try our tested condition first.

Our study focused on increasing the total amount of recombinant protein by enhancing the protein production per cell. Although the enzyme catalytic efficiency of several proteins was significantly higher than that in *E. coli*, it was also found that the shake-flask fermentation density of *V. natriegens* was 25% lower than that of *E. coli*, representing a possible bottleneck for industrial application. To solve the problem of low fermentation density, optimization can conceivably proceed in two aspects: (1) *Vibrio* sp. produce more extracellular polysaccharide and capsular polysaccharide than *E. coli* (Teschler et al., 2015). As a result, carbon sources are used for polysaccharide synthesis, which results in the cells being coated with capsular polysaccharides or biofilms, and the amount of extracellular polysaccharides in the fermentation broth increases (Flemming and Wingender, 2010). Blocking the polysaccharide synthesis pathway can redirect more carbon flux toward biomass accumulation and protein synthesis, and knocking out the polysaccharide synthesis pathway can also theoretically reduce the viscosity of the fermentation medium, thereby improving the mass transfer efficiency. (2) Knocking out the quorum-sensing system of *V. natriegens* may also increase the maximal cell density of the microbial population (Marrs and Swalla, 2008). The competitiveness of the *V. natriegens* protein expression system will be greatly improved if the number of cells per unit volume could be increased.

In conclusion, our study demonstrated that *V. natriegens* is an effective pET-based expression host complementary to *E. coli*. We hope that these findings will inspire more researchers to introduce their pET plasmids with poor expression in *E. coli* into *V. natriegens*. Furthermore, a consensus “what to try first” protocol was proposed to further enhance the protein production in *V. natriegens*, as well as providing guidance to researchers interested in *V. natriegens*. Finally, we also hope to inspire other researchers to further optimize this new bacterial chassis to make it much more competitive for recombinant protein expression.

## Materials and Methods

### Construction of Plasmids and Strains

The strains and plasmids used in this study are listed in Supporting information Supplementary Table 2, and the primers were listed in Supplementary Table 4.

All restriction enzymes used in this study were purchased from Thermo Fisher Scientific (Waltham, United States). The PrimeSTAR Max^®^ (DNA polymerase TaKaRa, China) and 2 × Rapid Taq Master Mix (Vazyme, China) were used for gene cloning and colony PCR, respectively.

The plasmid pMD19T-dnsaLR-T7RNApol was cloned in two steps: (1) The pMD19T vector was linearized by *Xba*I digestion. The spectinomycin cassette was cloned from pTargetF, and the 3-kb homologous arms were cloned from the genome of *Vibrio natriegens* ATCC14048. These three fragments were assembled with the linearized pMD19T by Gibson Assembly, yielding pMD19T-dnsaLR-spec. (2) The pMD19T-dnsaLR-spec plasmid was further digested with *Hin*dIII, and the larger DNA fragment was extracted using the AxyPrep DNA Gel Extraction Kit. The T7 RNA polymerase cassette was cloned from the genome of *E. coli* BL21(DE3), and the spectinomycin cassette was cloned from pMD19T-dnsaLR-spec. The three fragments were assembled by Gibson Assembly (Gibson et al., 2009), resulting in the plasmid pMD19T-dnsaLR-T7RNApol.

Plasmids were constructed and propagated in *E. coli* DH5α, purified using Miniprep Kits (Qiagen, Germany), and verified by Sanger sequencing (TsingKe, China). The plasmid pMD19T-dnsaLR-T7RNApol was then digested with *Xba*I, yielding the linearized DNA fragments dnsaLR-T7RNApol, which was used for further natural transformation. The *V. natriegens* strain VnDX, which was derived from ATCC 14048 by integrating the T7 RNA polymerase expression cassette at the *dns* locus, was constructed using MuGENT method (Dalia et al., 2017). Plasmids were transferred into *V. natriegens* using a previously published electroporation protocol (Weinstock et al., 2016).

### Media and Culture Conditions

The *E. coli* strains were grown in LB or TB medium at 37°C, supplemented where necessary with 100 μg/ml ampicillin (Amp) or 100 μg/ml kanamycin (StateKan). The *V. natriegens* strains were grown in LBv2, BHIv2 (Weinstock et al., 2016), or TBv2 medium (12 g/L tryptone, 24 g/L yeast extract, 0.5% v/v glycerol, 15 g/L NaCl, 2.31 g/L KH_2_PO_4_, 16.43 g/L K_2_HPO_4_⋅3H_2_O) medium at 30°C. Where needed, 15 g/L agar added to produce solid medium. Plasmids encoding *TfoX* were maintained in *V. natriegens* by adding 100 μg/ml carbenicillin (Carb). *V. natriegens* strains maintaining pET plasmids were supplied with 100 μg/ml Carb or 200 μg/ml StateKan.

### Shake-Flask Fermentation

*V. natriegens* strains harboring pET plasmids were grown in 5 ml of LBv2 liquid medium overnight at 30°C. Then, 300 μl of the resulting seed cultures were used to inoculate 30 ml of BHIv2 medium in 250-ml shake flasks. IPTG was added to a final concentration of 0.3 mM when seed cultures were inoculated, and fermentation was allowed to continue for an additional 22 h at 30°C.

*E. coli* strains harboring pET plasmids were grown in 5 ml of LB liquid medium overnight at 37°C. Then, 300 μl of the resulting seed cultures were used to inoculate 30 ml of TB medium in 250-ml shake flasks. Cells were cultured at 37°C to an OD_600_ of 2–4 (nearly 3 h), at which point IPTG was added to a final concentration of 0.3 mM, and the fermentation was allowed to continue at 28°C for an additional 22 h. The fermentation conditions for *E. coli* BL21(DE3) used in this study were optimized by our laboratory in earlier work (Sun et al., 2020; Zhang et al., 2020).

After 22-h fermentation, the cell density was measured by spectrophotometer at 600 nm. Then, 1 ml final fermentation culture was collected in 1.5-ml Eppendorf tubes, respectively, for subsequent protein expression analysis.

### Sodium Dodecyl Sulfate-Polyacrylamide Gel Electrophoresis Analysis

The collected 1-ml sample of the fermentation broth was centrifuged for 10 min at 12,000 rpm, and the supernatant was removed. The cells were resuspended in 1 ml of 20 mM Tris-HCl buffer (pH 7.5) and lysed using an Ultrasonic Cell Disruptor (operating for 5 s, pausing for 5 s, 40 times). The cell lysate was centrifuged at 4°C, 12,000 rpm for 10 min to remove the cell debris. Eighty microliters of the supernatant was mixed with 20 μl of 5 × SDS-PAGE loading buffer and heated to 100°C in a metal block for 10 min. The final protein samples were used for SDS-PAGE (WSHT, China).

To facilitate the gel analysis, we calculated normalized loading volumes for standard gels (Novagen). Samples containing the protein from an amount of cells corresponding to 0.15 OD_600_ units (10-well gel) or 0.075 OD_600_ units (15-well gel) were added to the corresponding protein gel wells and separated at 90 V until the dye reached at the end of the gel. Quantitative comparison was conducted using Quantity One 1-D software (BioRad, United States) and Quantity One User Guide for Version 4.6.2. According to the calculation results of Quantity One, *N*_*Vn*_ represents the value of the GOI overexpression band in VnDX, and *N*_*Ec*_ represents the value of the GOI overexpression band in BL21(DE3). VnDX = BL21(DE3) when the following inequality is satisfied. GraphPad prism software was used for statistical analysis of data.

$$\left|\frac{N_{Vn}-N_{Ec}}{N_{Ec}}\right|\leq 0.1$$

### Glucose Dehydrogenase Activity Assay

To measure GDH activity, 1 ml of fermentation broth was treated as described above, and the resulting supernatant was the crude enzyme solution. The reaction mixture contained 100 mM glucose, 100 mM Tris-HCl (pH 7.5), 2 mM NAD^+^, and the diluted crude enzyme solution. The GDH activity was assayed at 30°C by following the increase of the absorbance at 340 nm (A_340_) due to the reduction of NAD^+^ to NADH. The enzyme activity was calculated using the formula U/ml = [ΔA/min] × [1/ε] × 8,000 [ε = A/cL = 6.402 ml/(μmol × cm)] (Bucher et al., 1974).

### Carbonyl Reductase Activity Assay

To measure carbonyl reductase activity, 1 ml of fermentation broth was treated as described above, and the resulting supernatant was the crude enzyme solution. The reaction mixture contained 16 g/L isopropanol, 100 mM Tris-HCl (pH 7.5), 2 mM NAD^+^, and the diluted crude enzyme solution. The carbonyl reductase activity was assayed at 37°C by following the increase ofA_340_. The enzyme activity was calculated using the formula U/ml = [ΔA/min] × [1/ε] × 8,000 [ε = A/cL = 6.402 ml/(μmol × cm)] (Bucher et al., 1974).

### D-Amino Acid Oxidase Activity Assay

To measure D-amino acid oxidase activity, 1 ml of fermentation broth was treated as described above, and the resulting supernatant was the crude enzyme solution. Then, 0.1 ml of the diluted crude enzyme was pipetted into a 1.5 cm × 15 cm test tube, and 400 IU of catalase (Sigma-Aldrich) and 1 ml 0.1 M D-alanine [prepared with 0.1 M phosphate buffered saline (PBS), pH 8.0, Sangon Biotech, China] were added. The mixed solution was placed in a water bath at 37°C and shaken at 180 rpm for 10 min. Then, 0.6 ml of 10% trichloroacetic acid (Aladdin, China) was added to terminate the reaction and 0.1 ml of the mixed solution was diluted with 0.9 ml PBS, followed by the addition of 0.4 ml of 0.2% 2.4-dinitrophenylhydrazine (Aladdin, China) and incubation for 10 min at room temperature (RT). Then, 1.5 ml 3 M NaOH (Sangon Biotech, China) was added, mixed well, and incubated for 15 min at RT. The mixture was then centrifuged at 8,000 rpm for 5 min, and the supernatant was taken to measure the absorbance at 550 nm (Zheng et al., 2007). One unit (U) of DAAO enzyme activity was defined as the amount of enzyme required to produce 1 μmol of sodium pyruvate per minute.

## Data Availability Statement

The raw data supporting the conclusions of this article will be made available by the authors, without undue reservation.

## Author Contributions

JX, FD, JY, YJ, and SY developed the research plan. JX, FD, and MW performed the experiments. JX and RT collected and analyzed data. JX, JY, MW, YJ, SY, and LY wrote the manuscript. All authors commented on and revised the manuscript.

## Conflict of Interest

RT was employed by company Huzhou Yisheng Biotechnology Co., Ltd. YJ was employed by company Shanghai Taoyusheng Biotechnology Co., Ltd. The remaining authors declare that the research was conducted in the absence of any commercial or financial relationships that could be construed as a potential conflict of interest.

## Supplementary Material

Supplementary Figure 1 Construction of *V. natriegens* VnDX strain and GFP expression using pET-based system. **(A)** Schematic of the construction of VnDX. The T7 RNA polymerase and specR cassettes were integrated into the *dns* locus using natural transformation (MuGNET described in section “Materials and Methods”). tDNA, transformed DNA. **(B)** plasmid pET-24a-GFP was transformed into VnDX and BL21(DE3), respectively, and GFP fluorescence was detected with or without IPTG in liquid medium. Top: cells under white light; bottom: cells under blue light. VnDX or BL21(DE3) strain containing pET-24a-GFP showed visible GFP fluorescence after IPTG induction. **(C)** GFP fluorescence was detected with or without IPTG on LBv2 agar plate. Top: cells under white light; bottom: cells under blue light. 1#: *V. natriegens*/pET-24a; 2#: VnDX/pET-24a; 3#: *V. natriegens*/pET-24a-GFP; 4#: VnDX/pET-24a-GFP; 5#: BL21(DE3)/pET-24a-GFP. Only *V. natriegens* strain with T7 RNA polymerase cassette and pET-24a-GFP showed GFP fluorescence after IPTG induction. **(D)** SDS–PAGE analysis of soluble cytosolic fraction of lysed cells (the GFP band is marked with a red arrow).

Supplementary Figure 2 Comparison the soluble expression of 196 GOI in our library according to SDS-PAGE. **(A–T)** 0.15 OD (10-well gel) or 0.075 OD (15-well gel) prepared crude protein samples were loaded to the corresponding protein gel wells. V, *V. natriegens* VnDX; E, *E. coli* BL21(DE3); Ctrl, Glucose dehydrogenase from *Bacillus subtillis* contained in each batch of fermentation as a positive control; Blue box, GOI possessing higher expression in VnDX than BL21(DE3); Orange box, GOI possessing the same expression in VnDX and BL21(DE3); Gray box, GOI possessing higher expression in BL21(DE3) than VnDX. Numbers above the lanes represent *V. natriegens* or *E. coli* strains and the detailed information of these strains were shown in Supplementary Table 3.

Supplementary Figure 3 Classifications of the evaluation results of these 196 GOI in VnDX and BL21(DE3) according to enzyme families, sources and lengths of amino acids. **(A)** Classification according to enzyme family. **(B)** Classification according to enzyme sources. **(C)** Classification according to the length of enzymes. VnDX>BL21(DE3): GOI possessing higher expression in VnDX than BL21(DE3); VnDX=BL21(DE3): GOI possessing the same expression in VnDX and BL21(DE3); VnDX<BL21(DE3): GOI possessing higher expression in BL21(DE3) than VnDX.

Supplementary Figure 4 Growth curve and protein expressions of four GOI in BHIv2, TBv2, and LBv2 culture medium. **(A)** Simvast acyltransferase from *Aspergillus terreus*. **(B)** D-amino acid transaminase from *Silicibacter promeroyi* DSS-3. **(C)** Haine racemase from *Agrobacterium tumefaciens str.* C58. **(D)** Maltooligosaccharide trehalose hydrolase from *Arthrobacterium* S 34. 0.15 OD_600_ (10-well gel) prepared crude protein samples were loaded to the corresponding protein gel wells. Error bars represent the SD of *n* = 3 technical replicates.

Supplementary Figure 5 The growth and GDH catalytic efficiency curve of VnDX/pGDH at 30 or 28°C. **(A)** the growth curve of VnDX/pGDH at 30 or 28°C. **(B)** the GDH catalytic efficiency (U/ml) curve of VnDX/pGDH at 30 or 28°C. Error bars represent the SD of *n* = 3 technical replicates.

Supplementary Figure 6 The GDH catalytic efficiency per OD_600_ (U⋅ml^–1^⋅OD^–1^) in BL21(DE3)/pGDH and VnDX/pGDH at the end of fermentation (24 h). Error bars represent the SD of *n* = 3 technical replicates.

Supplementary Figure 7 The growth curve of *V. natriegens* at 37 or 30°C. Error bars represent the SD of *n* = 3 technical replicates.

Supplementary Figure 8 The SDS-PAGE of soluble and insoluble expression of 3 GOI. 0.15 OD_600_ prepared crude protein samples were loaded to the corresponding protein gel wells. V, *V. natriegens* VnDX; E, *E. coli* BL21(DE3); Sol, soluble expression proteins; Insol, insoluble expression proteins. The GOI band is marked with red arrows. The detailed information of these GOI were shown in Supplementary Table 3.

Supplementary Table 1 Enzyme classification of 196 GOI in our library.

Supplementary Table 2 Strains and plasmids used in this work.

Supplementary Table 3 Detailed information of 196 GOI in *E. coli* and *V. natriegens*.

Supplementary Table 4 Primers used in this study.
