# Supplementary material for: Vibrio natriegens as a pET-Compatible Expression Host Complementary to Escherichia coli
Source: Front Microbiol. 2021 Feb 19;12:627181. doi: 10.3389/fmicb.2021.627181 (PMC7933001; doi:10.3389/fmicb.2021.627181)
Supplement: Supplementary Figure 1 — Construction of V. natriegens VnDX strain and GFP expression using pET-based system. (A) Schematic of the construction of VnDX. The T7 RNA polymerase and specR cassettes were integrated into the dns locus using natural transformation (MuGNET described in section “Materials and Methods”). tDNA, transformed DNA. (B) plasmid pET-24a-GFP was transformed into VnDX and BL21(DE3), respectively, and GFP fluorescence was detected with or without IPTG in liquid medium. Top: cells under white light; bottom: cells under blue light. VnDX or BL21(DE3) strain containing pET-24a-GFP showed visible GFP fluorescence after IPTG induction. (C) GFP fluorescence was detected with or without IPTG on LBv2 agar plate. Top: cells under white light; bottom: cells under blue light. 1#: V. natriegens/pET-24a; 2#: VnDX/pET-24a; 3#: V. natriegens/pET-24a-GFP; 4#: VnDX/pET-24a-GFP; 5#: BL21(DE3)/pET-24a-GFP. Only V. natriegens strain with T7 RNA polymerase cassette and pET-24a-GFP showed GFP fluorescence after IPTG induction. (D) SDS–PAGE analysis of soluble cytosolic fraction of lysed cells (the GFP band is marked with a red arrow). [file Data_Sheet_1.docx]

**Figure S1. Construction of *Vibrio natriegens* VnDX strain and GFP expression using pET-based system**


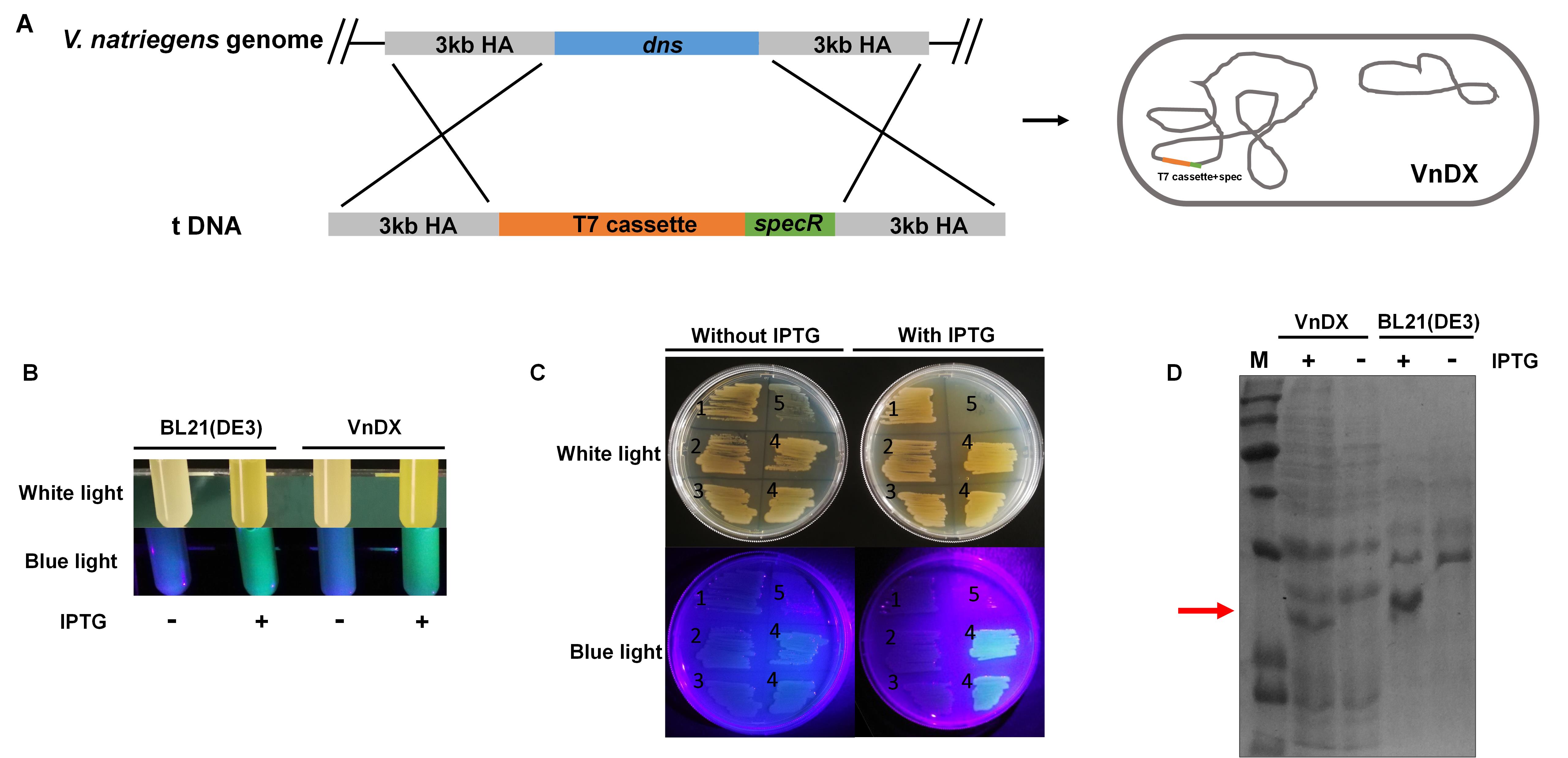


**Figure S2. Comparison the soluble expression of 196 GOI in our library according to SDS-PAGE**























**

**

**

**


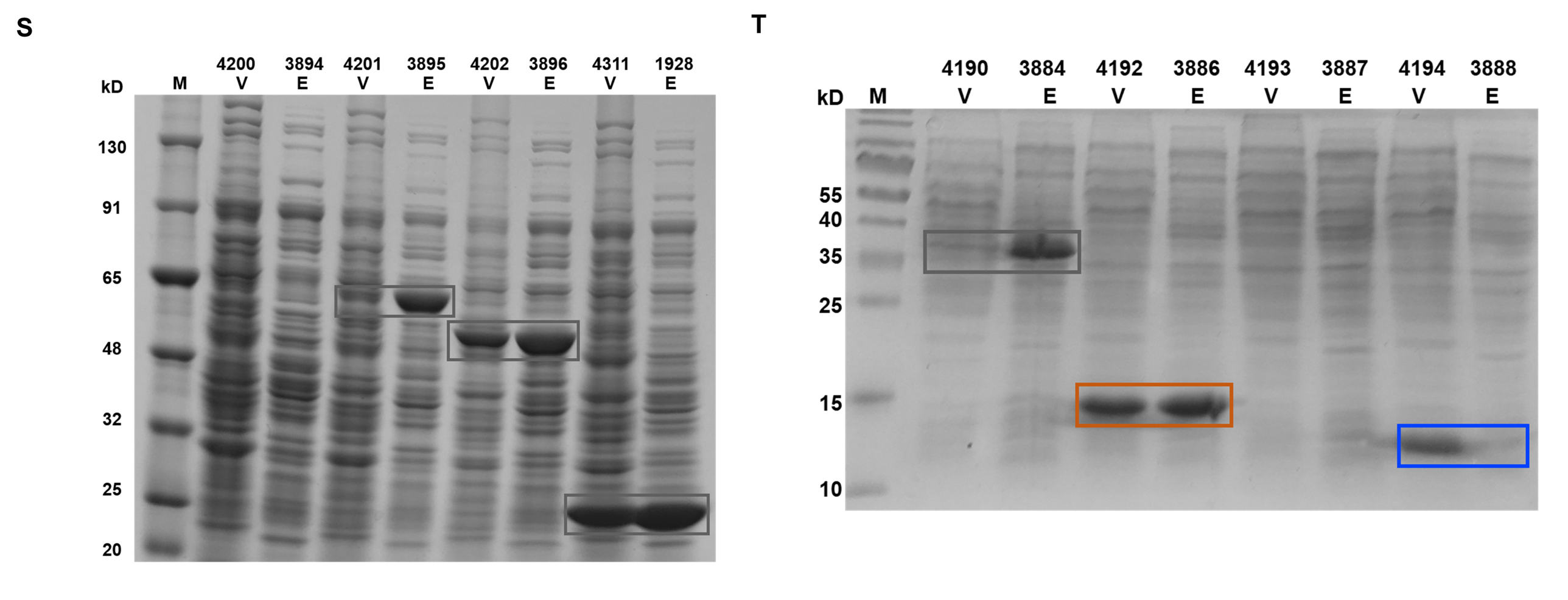


**Figure S3. Classifications of the evaluation results of these 196 GOI in VnDX and BL21(DE3) according to enzyme families, sources and lengths of amino acids**





**Figure S4. Growth curve and protein expressions of four GOI in BHIv2, TBv2 and LBv2 culture medium**





**Figure S5. The growth and GDH catalytic efficiency curve of VnDX/pGDH at 30 ℃ or 28 ℃.**


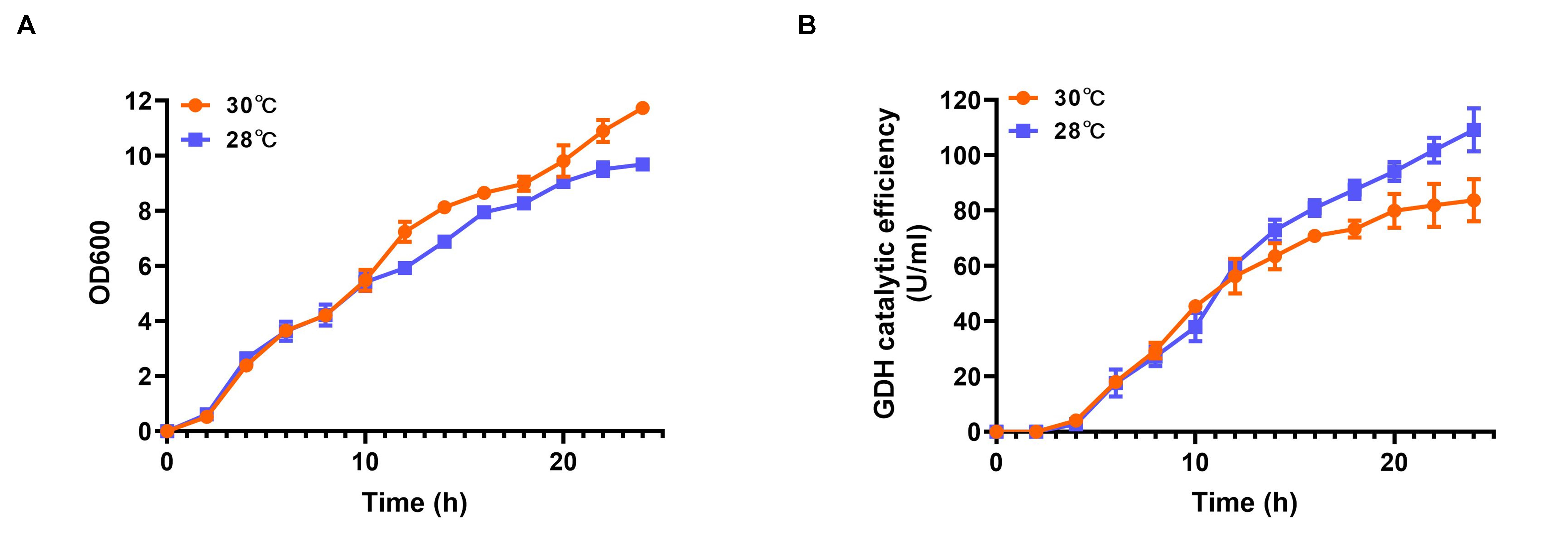


**Figure S6. The** **GDH catalytic efficiency per OD_600_ (U·ml^-1^·OD^-1^) in BL21(DE3)/pGDH and VnDX/pGDH at 24 h**


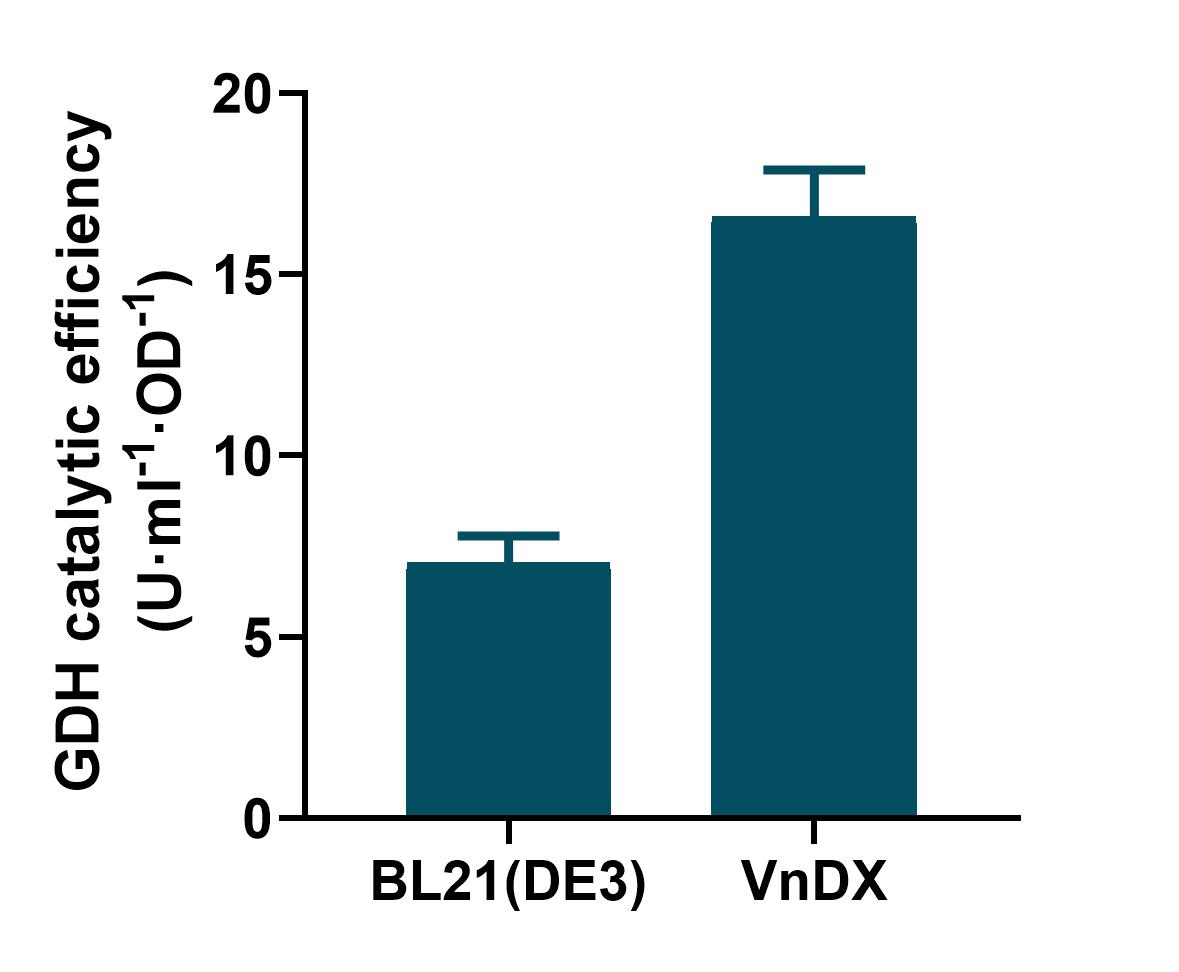


**Figure S7. The growth curve of *V. natriegens* at 37 ℃ or 30 ℃**

**
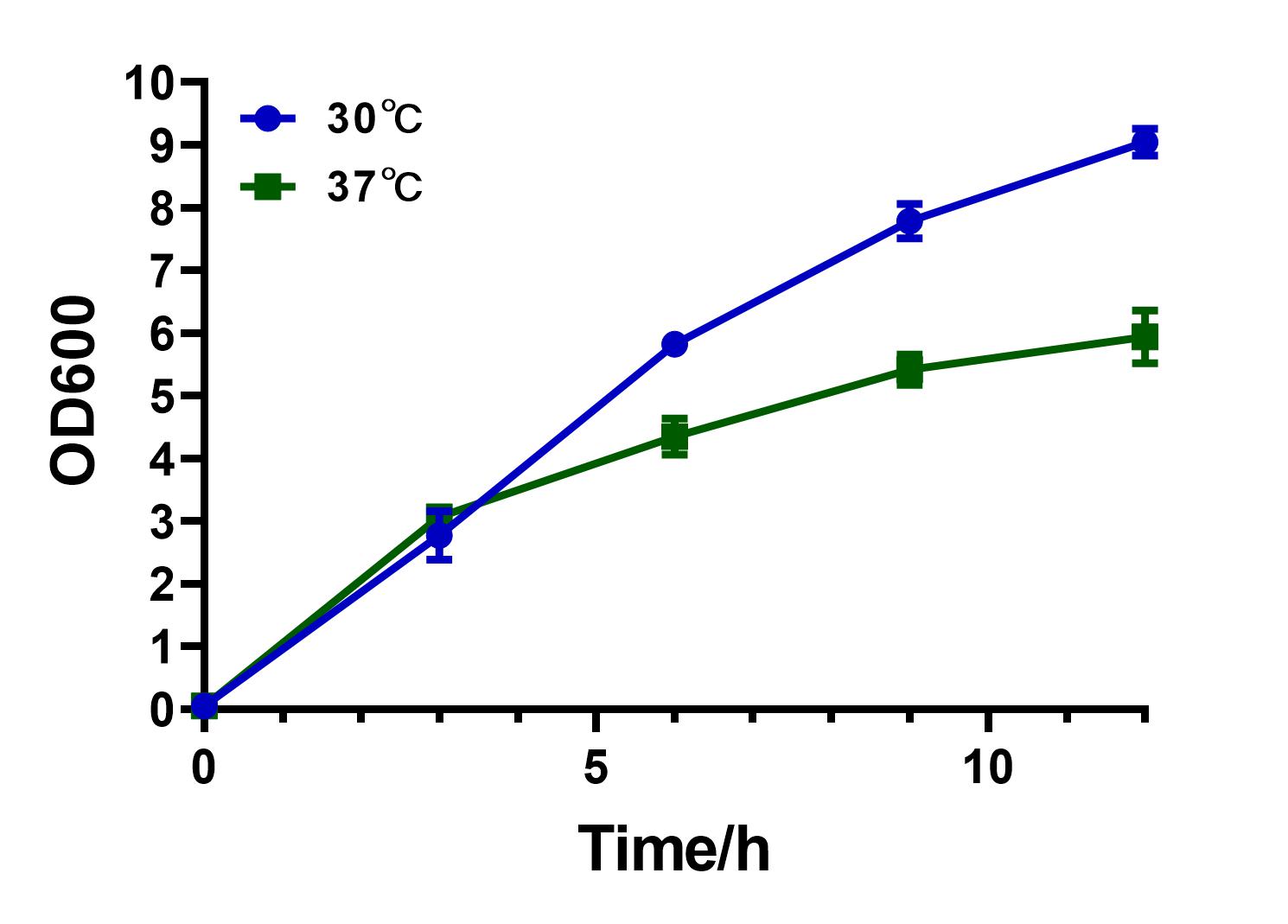
**

**Figure S8. The SDS-PAGE of soluble and insoluble expression of 3 GOI.**





**Table S1 Enzyme classification of 196 GOI in our library.**

| **Enzyme classification** | **Examples** | **Amount** |
| --- | --- | --- |
| Oxidoreductase | glucose dehydrogenase, carbonyl reductase, amino acid oxidase | 62 |
| Transferase | glycosyltransferase, aminotransferase | 53 |
| Hydrolase | lactamase, nitrilase, penicillin acylase | 30 |
| Lyase | aldolase, nitrile hydratase | 29 |
| Isomerase | alanine racemase, fructose isomerase | 15 |
| Ligase | glutathione synthetase, CTP synthetase | 6 |
| Translocase | K+ transporter | 1 |
| **Total** |  | **196** |

**Table S2 Strains and plasmids used in this work. All primers are synthesized by TsingKe Co., Ltd., China.**

| **Strains or plasmids** | **Description** | **Source or reference** |
| --- | --- | --- |
| **Strains** |  |  |
| *Vibrio natriegens* ATCC14048 | Wild Type | ATCC |
| *Escherichia coli* DH5α | F-, φ80dlacZΔM15, Δ(lacZYA-argF)U169, deoR, recA1, endA1, hsdR17(rk-, mk), phoA, supE44, λ-, thi-1, gyrA96, relA1 | Takara |
| *Escherichia coli* BL21(DE3) | F-, ompT, hsdSB(rBB-mB-), gal, λ(DE3 [lacI lacUV5-T7p07 ind1 sam7 nin5]) [malB+]K-12(λS) | Thermo Fisher Scientific |
| VnDX | *Vibrio natriegens* ATCC14048 ∆dns::(lacI-lacUV5-lacO-T7RNA polymerase-spec^R^) carrying pMMB67EH-tfoX | This study |
| VnDX/pET-24a-GFP | VnDX carrying pET-24a-GFP | This study |
| *V. natriegens*/pET-24a-GFP | *Vibrio natriegens* ATCC14048 carrying pET-24a-GFP | This study |
| *V. natriegens*/pET-24a | *Vibrio natriegens* ATCC14048 carrying pET-24a | This study |
| VnDX/pET-24a | VnDX carrying pET-24a |  |
| BL21(DE3)/pGFP | BL21(DE3) carrying pET-24a-GFP | This study |
| VnDX/pGDH | VnDX carrying pET-24a-GDH | This study |
| BL21(DE3)/pGDH | BL21(DE3) carrying pET-24a-GDH | This study |
| Table S3 | 196 VnDX and BL21(DE3) strains containing different pET plasmids encoding corresponding enzymes | This study |
| **Plasmids** |  |  |
| pTargetF | Plasmid carrying spectinomycin resistance cassette | Addgene 62226 |
| pET-24a | Kan, T7 lac promoter, his-tag | Novagen |
| pMD19T | Plasmid carrying ampicillin resistance cassette and pUC origin | Takara |
| pMMB67EH-tfoX | pMMB67EH harboring pTac promoter and *tfoX* gene from *vibrio cholerae* | (Dalia, Hayes et al. 2017) |
| pET-24a-GFP | pET-24a carrying GFP expression cassette | (Zhang et al. 2020) |
| pET-24a-GDH | pET-24a carrying *Bacillus subtillis* glucose dehydrogenase expression cassette | (Zhang et al. 2020) |
| pMD19T-dnsaLR-spec | pMD19TSimple carrying spectinomycin resistance cassette and 3kb homologous arms flank *dns* locus | This study |
| pMD19T-dnsaLR-T7RNApol | pMD19TSimple carrying lacI-lacUV5-lacO-T7 RNA polymerase cassette, spectinomycin resistance cassette and 3kb homologous arms flank *dns* locus | This study |

**Table S3. Detailed information of 196 GOI in *E. coli* and *V. natriegens***

| **Name** | **Source** | **Plasmid** | **Strain number of BL21(DE3)** | **Strain**  **number of VnDX** | **Evaluating the GOI expression in VnDX and BL21(DE3)** | | | |  |
| --- | --- | --- | --- | --- | --- | --- | --- | --- | --- |
|  |  |  |  |  | **VnDX＞BL21(DE3)** | **VnDX＝BL21(DE3)** | **VnDX＜BL21(DE3)** | **no obvious overexperssion band in either organism** | **Reference** |
| Secondary alcohol dehydrogenase (NADH regeneration) | *B.stearothermophilus* (NCIMB 12403) | pET28b-ADH-ht | CIBT3787 | CIBT3977 |  |  | ⁕ |  | NCBI: CAA81612 |
| Secondary alcohol dehydrogenase (NADH regeneration) | *Rhodococcus Ruber* | pET24a-ADH-A | CIBT3788 | CIBT3978 |  |  |  | ⁕ | PDB: 3JV7_A |
| Secondary alcohol dehydrogenase (NADPH regeneration) | *T.brockii* | pET24a-tbADH | CIBT3789 | CIBT3979 |  | ⁕ |  |  | NCBI: CAA46053 |
| Secondary alcohol dehydrogenase (NADPH regeneration) | *Bacillus sp.* ECU0013 | pET24a-BsADH | CIBT3790 | CIBT3980 |  |  | ⁕ |  | Y Ni, et al. Appl Microbiol Biotechnol 2011, 89(4): 1111-1118 |
| R-carbonyl reductase (NAD regeneration) | *Ogataea str.* | pET24a-CIR | CIBT3791 | CIBT3981 |  |  | ⁕ |  | CN1993464B |
| S-carbonyl reductase (NAD regeneration) | *Candida parapsilosis* | pET24a-CpSADH | CIBT3792 | CIBT3982 |  |  |  | ⁕ | NCBI: BAA24528 |
| S-carbonyl reductase (NAD regeneration) | *Leifsonia sp.* S749 | pET24a-LSADH | CIBT3793 | CIBT3983 | ⁕ |  |  |  | GenBank: BAD99642.1 |
| S-carbonyl reductase (NAD regeneration) | *Candida magnoliae* | pET24a-krh215 | CIBT3794 | CIBT3984 | ⁕ |  |  |  | WO2004/015132 A2 |
| S-carbonyl reductase (NAD regeneration) | *candida magnoliae* ifo 0705 | pET24a-CmADH | CIBT3795 | CIBT3985 | ⁕ |  |  |  | patent US6645746 |
| R-carbonyl reductase (NADP regeneration) | *Lactobacillus brevis* | pET24a-LbAdh | CIBT3796 | CIBT3986 |  |  | ⁕ |  | AJ544275.1 |
| R-carbonyl reductase (NADP regeneration) | *candida magnoliae* ifo 0705 | pET24a-CRHZ | CIBT3797 | CIBT3987 |  | ⁕ |  |  | mstplnalvtgasrgigaataiklaengysvtlaarnvaklnevkeklpvvkdgqkhhiweldlasveaassfkgaplpasdydlfvsnagiaqftptadqtdkdflniltvnlsspialtkallkgvsersnekpfhiiflssaaalhgvpqtavysaskagldgfvrslarevgpkgihvnvihpgwtktdmtdgiddpndtpikgwiqpeaiadavvflaksknitgtnivvdnglla |
| R-carbonyl reductase (NADP regeneration) | *Candida magnoliae* | pET24a-CmAdh3 | CIBT3798 | CIBT3988 |  |  | ⁕ |  | CN104830921A |
| R-carbonyl reductase (NADP regeneration) | *Candida magnoliae* | pET24a-CmAdh4 | CIBT3799 | CIBT3989 |  |  | ⁕ |  | GenBank: ABB91668.1 |
| R-carbonyl reductase (NADP regeneration) | *Novosphingobium aromaticivorans* DSM 12444 (G145A/I199L) (WO2005018579) | pET24a-KRED6 | CIBT3800 | CIBT3990 | ⁕ |  |  |  | WO2005018579 |
| R-carbonyl reductase (NADP regeneration) | *Saccharomyces cerevisiae* | pET24a-AH02 | CIBT3801 | CIBT3991 |  |  | ⁕ |  | msfhqqfftlnngnkipaiaiigtgtawykpeetdatfsnslveqivyalklpgiihidaaewyrtypevgkalsltekprnaifltdkyspqikmsdspadgldlalkkmgtdyvdlylihsplvskevnglsleeawkdmeqlyksgkaknigvsnftvedlqrilkvaevkpqvnqiefspflqnqtpgickfcqehdilveaysplgplrkktaqddsqpffeyvkelsekynkseaqiilrwvtkrgvlpvttsskpqrisdaqnlfsfdltaeevdkitelgleheplrlywnklygkynyaaqkv |
| R-carbonyl reductase (NADP regeneration) | WO 2008/042876 A2 | pET24a-KRED326 | CIBT3802 | CIBT3992 |  |  | ⁕ |  | WO2008/042876 A2 |
| R-carbonyl reductase (NADP regeneration) | *Acinetobacter baylyi* | pET24a-dkr | CIBT3803 | CIBT3993 | ⁕ |  |  |  | WP_021765610.1 |
| R-carbonyl reductase (NADP regeneration) | *Leuconostoc mesenteroides subsp.* | pET24a-AR | CIBT3804 | CIBT3994 |  |  | ⁕ |  | mkiaiagfgalgarlgvmlqagghevtgidgwpahiaaintkgltvvkdndapqkyfvpvmpasevtgtfdliilltktpqldrmltdiqpiitdttkllvlsnglgnievmakhvsrhqilagvtlwtsslikpgeihvtgsgsiklqaigdadvqsiadalnqaglnaeitpdvmtaiwhkaginavlnplsvllnaniaefgtagnamdlalnildemkqvgasqgikvdvsgimtdlsqllkpenagnhfpsmyqdiqngkrteidflngyfakighesgiptpfnalvtrlihakediervklakqqenfei |
| S-carbonyl reductase (NADP regeneration) | *Lactobacillus kefiri* DSM 20587 (WO2010025238) | pET24a-adhR60 | CIBT3805 | CIBT3995 |  | ⁕ |  |  | WO2010025238 |
| S-carbonyl reductase (NADP regeneration) | CN 101889081 A：No.20 | pET24a-KRED20 | CIBT3806 | CIBT3996 |  |  | ⁕ |  | Montelukast (CN101889081A：No.20) |
| S-carbonyl reductase (NADP regeneration) | *Lactobacillus kefiri* | pET24a-KRED38 | CIBT3807 | CIBT3997 | ⁕ |  |  |  | mtdrlkgkvaivtggtlgiglaiadkfveegakvvitgrhadvgekaaksiggtdvirfvqhdasdeagwtklfdtteeafgpvttvvnnagiavsksvedttteewrkllsvnldgvffgtrlgiqrmknkglgasiinmssieglvgdptlgaynaskgavrimsksaaldcalkdydvrvntvhpgpiktpllddlegweemmsqrtktpmghigepndiawicvylasdeskfatgaefvvdggytaq |
| Alcohol Dehydrogenase | *Thermoplasma acidophilum* DSM 1728 | pET28b-Ta1316 | CIBT3808 | CIBT3998 |  |  |  | ⁕ | WP_010901722.1 |
| Lactic dehydrogenase | *T.maritima* | pET24a-LDH | CIBT3809 | CIBT3999 |  |  | ⁕ |  | WP_004082418.1 |
| 6-phosphogluconic dehydrogenase | *E. coli* | pET24a-gnd | CIBT1924 | CIBT4307 |  |  |  |  | WP_000043484.1 |
| Glucose dehydrogenase | *Bacillus subtilis* | pET28b-GDH | CIBT3810 | CIBT4000 |  |  |  | ⁕ | CN104830921A |
| Glucose dehydrogenase | *T.acidophilum* | pET24a-TaGDH | CIBT3811 | CIBT4001 |  |  |  | ⁕ | GenBank: CAA42450.1 |
| glucose-6-phosphate dehydrogenase | *E. coli* | pET24a-zwf | CIBT1923 | CIBT4306 |  |  |  |  | WP_000301727.1 |
| Mannitol dehydrogenase | *Leuconostoc pseudomesenteroides* | pET24a-LpMDH | CIBT3812 | CIBT4002 |  |  | ⁕ |  | WP_010281376.1 |
| Mannitol dehydrogenase | *Thermotoga maritima* MSB8 | pET24a-TM0298 | CIBT3813 | CIBT4003 |  | ⁕ |  |  | WP_004083017.1 |
| L-2-hydroxy isohexyl dehydrogenase | *Lactobacillus confuse*s DSM 20196 | pET24a-L-HicDH | CIBT3814 | CIBT4004 |  | ⁕ |  |  | markigiiglgnvgaavahgliaqgvaddyvfidaneakvkadqidfqdamanleahgnivindwaaladadvvistlgniklqqdnptgdrfaelkftssmvqsvgtnlkesgfhgvlvvisnpvdvitalfqhvtgfpahkvigtgtlldtarmqravgeafdldprsvsgynlgehgnsqfvawstvrvmgqpivtladagdidlaaieeearkggftvlngkgytsygvatsairiakavmadahaelvvsnrrddmgmylsypaiigrdgvlaettldlttdeqekllqsrdyiqqrfdeivdtl |
| D-2-hydroxy isohexyl dehydrogenase | *Lactobacillus paracasei* DSM 20008 | pET28b-D-HicDH | CIBT3815 | CIBT4005 |  |  | ⁕ |  | WP_003577354.1 |
| Formate dehydrogenase | *Candida boidinii* | pET28b-CbFDH | CIBT3816 | CIBT4006 |  | ⁕ |  |  | GenBank: OWB83932.1 |
| Formate dehydrogenase | *Pseudomonas sp.* 101 | pET24a-PseFDH | CIBT3817 | CIBT4007 | ⁕ |  |  |  | R Tao et al. Biotechnol Lett 2014, 36(4): 835-841 |
| Formate dehydrogenase | *Candida methylica* | pET24a-CmFDH | CIBT3818 | CIBT4008 |  |  | ⁕ |  | GenBank: CAA57036.1 |
| Pyruvic oxidase | *E.coli* MG1655 | pET24a-poxB | CIBT3819 | CIBT4009 |  | ⁕ |  |  | WP_000815337.1 |
| Pyruvic oxidase | *Lactobacillus plantarum* | pET24a-lpPOX | CIBT3820 | CIBT4010 |  |  |  | ⁕ | mvmkqtkqtnilagaavikvleawgvdhlygipggsinsimdalsaerdrihyiqvrheevgamaaaadakltgkigvcfgsagpggthlmnglydaredhvpvlaligqfgttgmnmdtfqemnenpiyadvadynvtavnaatlphvideairrayahqgvavvqipvdlpwqqisaedwyasannyqtpllpepdvqavtrltqtllaaerpliyygigarkagkeleqlsktlkiplmstypakgivadrypaylgsanrvaqkpanealaqadvvlfvgnnypfaevskafkntryflqididpaklgkrhktdiavladaqktlaailaqvserestpwwqanlanvknwraylasledkqegplqayqvlravnkiaepdaiysidvgdinlnanrhlkltpsnrhitsnlfatmgvgipgaiaaklnyperqvfnlagdggasmtmqdlvtqvqyhlpvinvvftncqygfikdeqedtnqndfigvefndidfskiadgvhmqafrvnkieqlpdvfeqakaiaqhepvlidavitgdrplpaeklrldsamssaadieafkqryeaqdlqplstylkqfglddlqhqigqggf |
| Alanine dehydrogenase | *Geobacillus stearothermophilus* strain XL-65-6 | pET24a-alaD | CIBT3821 | CIBT4011 |  | ⁕ |  |  | WP_011232226.1 |
| Alanine dehydrogenase | *B.sphaericus* | pET24a-BsALDH | CIBT3822 | CIBT4012 | ⁕ |  |  |  | UniProtKB/Swiss-Prot: P17556.1 |
| Glutamate dehydrogenase | *Pseudomonas putida* | pET28a-GluDH-3M | CIBT3956 | CIBT4262 |  |  | ⁕ |  | X Yin et al. Catalysis Science & Technology 2020 |
| Valine dehydrogenase | *Streptomyces albus* | pET24a-Vdh | CIBT3823 | CIBT4013 |  |  | ⁕ |  | WP_029394572.1 |
| L-leucine Dehydrogenase | *Bacillus cereus* ATCC14579 | pET28b-14579LeuDH | CIBT3824 | CIBT4014 |  | ⁕ |  |  | R Tao et al. Biotechnol Lett 2014, 36(4): 835-841 |
| L-leucine Dehydrogenase | *B.stearothermophilus* | pET28b-BacLeuDH | CIBT3825 | CIBT4015 |  |  | ⁕ |  | GenBank: AAA22570.1 |
| L-leucine Dehydrogenase | *T.intermedius* IFO 14230 | pET24a-14230leuDH | CIBT3826 | CIBT4016 |  | ⁕ |  |  | UniProtKB/Swiss-Prot: Q60030.1 |
| L-phenylalanine dehydrogenase | *Thermoactinomyces intermedius* | pET24a-BBH | CIBT3827 | CIBT4017 |  |  | ⁕ |  | UniProtKB/Swiss-Prot: P22823.1 |
| L-phenylalanine dehydrogenase | *Bacillus sphaericus* | pET28b-PheDH | CIBT3828 | CIBT4018 |  |  | ⁕ |  | UniProtKB/Swiss-Prot: P23307.1 |
| L-amino acid oxidase | *Proteus myxofaciens* DSM 4482 | pET24a-LAAD | CIBT3829 | CIBT4019 |  |  |  | ⁕ | GenBank: AXQ04983.1 |
| D-amino acid oxidase | *Rhodotorula gracilis* | pET28b-RgDAAO | CIBT3830 | CIBT4020 |  |  | ⁕ |  | H Zheng et al. J Biotechnol 2007, 129(3): 400-405 |
| D-amino acid oxidase | *Trigonopsis variabilis* | pET28b-TvDAAO | CIBT3831 | CIBT4021 | ⁕ |  |  |  | H Zheng et al. J Biotechnol 2007, 129(3): 400-405 |
| Monoamine oxidase | CN102131813A-WO2010008828 | pET24a-maoN19 | CIBT3832 | CIBT4022 |  |  | ⁕ |  | CN102131813A-WO2010008828 |
| L-glutamic oxidase | *Streptomyces sp.* X-119-6 | pET24a-lgox | CIBT3833 | CIBT4023 |  |  | ⁕ |  | CN104109698A |
| Lysine oxidase | *Pseudomonas sp.* AIU 813 (C254I) | pET15b-LAAO | CIBT4289 | CIBT4276 |  |  |  |  | US9181574 |
| Dihydrofolate reductase | *E. coli* W3110 | pET28a-DIR | CIBT3834 | CIBT4024 |  |  | ⁕ |  | WP_000624375.1 |
| Catalase | *E. coli* | pET24a-katG | CIBT3835 | CIBT4025 |  | ⁕ |  |  | GenBank: EGI08378.1 |
| Catalase | *E. coli* | pET24a-katE | CIBT3836 | CIBT4026 |  |  | ⁕ |  | PDB: 4ENS_A |
| Catalase | *Methanobrevibacter arboriphilus* | pET24a-MaKat | CIBT3837 | CIBT4027 |  |  | ⁕ |  | GenBank: CAC28086.1 |
| Catalase | *Bordetella pertussis Tohama* I | pET24a-katA | CIBT3838 | CIBT4028 |  |  | ⁕ |  | GenBank: ETH04976.1 |
| Cyclohexanone monooxygenase | WO2011071982A2 SEQ 124 | pET24a-CHMO124 | CIBT3839 | CIBT4029 |  | ⁕ |  |  | Esomeprazole (WO2011071982A2 SEQ 124) |
| Cyclohexanone monooxygenase | WO2011071982A2 SEQ 158 | pET24a-CHMO158 | CIBT3840 | CIBT4030 |  |  | ⁕ |  | Esomeprazole (WO2011071982A2 SEQ 158) |
| Methylxanthine N1 demethylase | *Pseudomonas putida* | pET24a-ndmA-OPT | CIBT3841 | CIBT4031 |  |  |  | ⁕ | WP_080957099.1 |
| Methylxanthine N1 demethylase | *Pseudomonas putida* | pET24a-ndmD-OPT | CIBT3842 | CIBT4032 |  |  |  | ⁕ | UniProtKB/Swiss-Prot: H9N291.1 |
| Phenylalanine hydroxylase | *Chromobacterium violaceum* | pET24a-PAHD | CIBT3843 | CIBT4033 |  |  | ⁕ |  | D Chen et al. J Biol Chem 1998, 273(40):25594-25601 |
| Phosphite dehydrogenase | *Pseudomonas stutzeri* | pET24a-Opt12 | CIBT3844 | CIBT4034 |  |  | ⁕ |  | R Woodyer et al. Biochemistry 2003, 42(40): 11604-11614 |
| L-serine hydroxymethyltransferase | *E. coli* W3110 | pET28a-L-SerHy | CIBT3845 | CIBT4035 |  | ⁕ |  |  | WP_000919159.1 |
| Acetolactate Synthase | *Bsubtilis* 168 | pet28b-alsS* | CIBT3846 | CIBT4036 |  |  | ⁕ |  | L Zhu et al. Appl Microbiol Biotechnol 2011, 90(3): 903-910 |
| Simvast acyl transferase | *Aspergillus terreus* | pET24a-SIM | CIBT3847 | CIBT4037 |  |  | ⁕ |  | PDB: 4LCM_A |
| Cyclodextrin glycosyltransferase | *Geobacillus stearothermophilus* | pET28b-GsCgt | CIBT3848 | CIBT4038 |  | ⁕ |  |  | MGAGNLNKVNFTSDVVYQIVVDRFVDGNTSNNPSGALFSSGCTNLRKYCGGDWQGIINKINDGYLTDMGVTAIWISQPVENVFSVMNDASGSASYHGYWARDFKKPNPFFGTLSDFQRLVDAAHAKGIKVIIDFAPNHTSPASETNPSYMENGRLYDNGTLLGGYTNDANMYFHHNGGTTFSSLEDGIYRNLFDLADLNHQNPVIDRYLKDAVKMWIDMGIDGIRMDAVEHMPFGWQKSLMDEIDNYRPVFTFGEWFLSENEVDANNHYFANESGMSLLDFRFGQKLRQVLRNNSDNWYGFNQMIQDTASAYDEVLDQVTFIDNHDMDRFMIDGGDPRKVDMALAVLLTSRGVPNIYYGTEQYMTGNGDPNNRKMMSSFNKNTRAYQVIQKLSSLRRNNPALAYGDTEQRWINGDVYVYERQFGKDVVLVAVNRSSSSNYSITGLFTALPAGTYTDQLGGLLDGNTIQVGSNGSVNAFDLGPGEVGVWAYSATESTPIIGHVGPMMGQVGHQVTIDGEGFGTNTGTVKFGTTAANVVSWSNNQIVVAVPNVSPGKYNITVQSSSGQTSAAYDNFEVLTNDQVSVRFVVNNATTNLGQNIYIVGNVYELGNWDTSKAIGPMFNQVVYSYPTWYIDVSVPEGKTIEFKFIKKDSQGNVTWESGSNHVYTTPTNTTGKIIVDWQN |
| N-Acetylglucosamine transferase | *Saccharomyces cerevisiae* | pET24a-ScGNA1 | CIBT3849 | CIBT4039 |  |  |  | ⁕ | NP_009626 |
| Trehalose synthase | *Arthrobacter sp.* | pET24a-treY | CIBT3850 | CIBT4040 |  |  | ⁕ |  | UniProtKB/Swiss-Prot: Q44315.1 |
| Trehalose synthase | *Thermus thermophilus* | pET24a-treS | CIBT3851 | CIBT4041 |  |  |  | ⁕ | UniProtKB/Swiss-Prot: O06458.1 |
| Trehalose synthase | *Thermus caldophilus GK24* | pET24a-TcTre | CIBT3852 | CIBT4042 |  |  | ⁕ |  | GenBank: AAD50660.3 |
| Orotate phosphoribosyltransferase | *E. coli* | pET24a-pyrE | CIBT1926 | CIBT4309 |  |  |  |  | AAN82902.1 |
| Glycosyltransferase | *Sulfolobus solfataricus* | pET28b-GT | CIBT3853 | CIBT4043 |  |  |  | ⁕ | GenBank: BAA11008.1 |
| Aspartate transaminase | *Escherichia coli* | pET24a-aspC | CIBT3854 | CIBT4044 |  |  | ⁕ |  | WP_000462687.1 |
| R-ω-transaminase | *Arthrobacter sp.* KNK168 | pET24a-STTA | CIBT3855 | CIBT4045 |  |  | ⁕ |  | PDB: 5FR9_A |
| R-ω-transaminase | US20100285541A1 | pET24a-ATA117 | CIBT3856 | CIBT4046 |  | ⁕ |  |  | US20100285541A1 |
| S-ω-transaminase | *Vibrio fluvialis* (US2013089898A1 seq no.31) | pET24a-VfTA31 | CIBT3857 | CIBT4047 |  |  | ⁕ |  | US2013089898A1 seq no.31 |
| GABA-α-ketoglutaric acid transaminase | *E. coli* W3110 | pET28a-gabT | CIBT3955 | CIBT4261 |  |  | ⁕ |  | L Meng et al. Catalysis Letters 2018, 148(11): 3309-3314 |
| D-amino acid transaminase | *Lysinibacillus sphaericus* DSM28 | pET28b-LsTA | CIBT3858 | CIBT4048 |  |  | ⁕ |  | AVK95843.1 |
| D-amino acid transaminase | *Chloroflexus aurantiacus* DSM635 | pET24a-CauTA | CIBT3859 | CIBT4049 |  | ⁕ |  |  | WP_012256373.1 |
| D-amino acid transaminase | *Planctomyces maris* DSM8797 | pET24a-PmTA | CIBT3860 | CIBT4050 |  | ⁕ |  |  | WP_002644666.1 |
| D-amino acid transaminase | *Chloroflexus aggregans* DSM9485 | pET24a-CagTA | CIBT3861 | CIBT4167 |  |  | ⁕ |  | WP_015940596.1 |
| D-amino acid transaminase | *Pseudomonas putida* LW-4 | pET24a-PpTA | CIBT3862 | CIBT4168 |  |  |  | ⁕ | WP_016497525.1 |
| D-amino acid transaminase | *Silicibacter pomeroyi* DSS-3 genome | pET24a-SpgTA | CIBT3863 | CIBT4169 |  |  | ⁕ |  | WP_011049284.1 |
| D-amino acid transaminase | *Silicibacter pomeroyi* DSS-3 large plasmid | pET24a-SppTA | CIBT3864 | CIBT4170 |  |  | ⁕ |  | WP_011242053.1 |
| Alanine-valine transaminase | *E. coli* W3110 | pET24a-avtA | CIBT3865 | CIBT4171 |  |  | ⁕ |  | WP_000144361.1 |
| Alanine-valine transaminase | *E. coli* W3110 | pET28b-ilvE | CIBT3866 | CIBT4172 | ⁕ |  |  |  | WP_000208520.1 |
| S-ω-amino acid transaminase | *Aquamicrobium defluvii* | pET24a-ADTA | CIBT3867 | CIBT4173 |  | ⁕ |  |  | NCBI: EXL10521 |
| S-ω-amino acid transaminase | *Bradyrhizobium sp*. Ec3.3 | pET24a-BSTA | CIBT3868 | CIBT4174 |  |  | ⁕ |  | WP_027523883 |
| S-ω-amino acid transaminase | *Brucella neotomae* | pET24a-BNTA | CIBT3869 | CIBT4175 |  | ⁕ |  |  | WP_004687895 |
| S-ω-amino acid transaminase | *Novosphingobium acidiphilum* | pET24a-NATA | CIBT3870 | CIBT4176 |  | ⁕ |  |  | WP_028641684 |
| S-ω-amino acid transaminase | *Pseudaminobacter salicylatoxidans* | pET24a-PSTA | CIBT3871 | CIBT4177 |  |  | ⁕ |  | WP_019171585 |
| S-ω-amino acid transaminase | *Brucella abortus* | pET24a-BATA | CIBT3872 | CIBT4178 |  | ⁕ |  |  | WP_006212579 |
| S-ω-amino acid transaminase | *Paracoccus denitrificans* PD122 | pET24a-PDTA | CIBT3873 | CIBT4179 |  |  | ⁕ |  | ABL72050 |
| S-ω-amino acid transaminase | *Aspergillus terreus* NIH2624 | pET24a-AT-ωTA | CIBT3874 | CIBT4180 |  |  | ⁕ |  | XP_001209325.1 |
| S-ω-amino acid transaminase | *Hyphomonas neptunium* ATCC 15444 | pET24a-HN-ωTA | CIBT3875 | CIBT4181 |  |  | ⁕ |  | WP_148205885.1 |
| S-ω-amino acid transaminase | *Arthrobacter sp.* | pET24a-ArR-ωTA | CIBT3876 | CIBT4182 | ⁕ |  |  |  | PDB: 3WWH_A |
| S-ω-amino acid transaminase | *Ochrobactrum anthropi* ATCC 49188 | pET24a-OA-ωTA | CIBT3877 | CIBT4183 |  |  | ⁕ |  | WP_011982390.1 |
| Glucokinase | *E. coli* | pET24a-glk | CIBT1922 | CIBT4305 |  |  | ⁕ |  | WP_000170346.1 |
| NAD kinase | *Mycobacterium tuberculosis* | pET24a- ppnk | CIBT3878 | CIBT4184 |  |  |  | ⁕ | WP_003408383.1 |
| Choline kinase | *Saccharomyces cerevisiae* | pET28aCKI | CIBT3879 | CIBT4185 |  |  |  | ⁕ | Y Liu et al. Appl Microbiol Biotechnol 2017, 101(4): 1409-1417 |
| Acetate kinase | *Lactobacillus sanfranciscensis* | pET24a-ack | CIBT3880 | CIBT4186 |  |  |  | ⁕ | UniProtKB/Swiss-Prot: Q9F1X7.1 |
| Acetate kinase | *Thermotoga maritima* MSB8 | pET24a-ackA | CIBT3881 | CIBT4187 |  |  | ⁕ |  | WP_004082978.1 |
| Acetate kinase | *E. coli* | pET24a-Eco-ack | CIBT3882 | CIBT4188 | ⁕ |  |  |  | Y Liu et al. Appl Microbiol Biotechnol 2017, 101(4): 1409-1417 |
| Aspartokinase | *Streptococcus pneumoniae* | pET24a-strpnlysC | CIBT3883 | CIBT4189 |  |  | ⁕ |  | WP_000869679.1 |
| Polyphosphate kinase | *Rhodobacter sphaeroides* | pET24a-RsPPK | CIBT3884 | CIBT4190 |  |  | ⁕ |  | WP_011338472.1 |
| Polyphosphate kinase | *E. coli* | pET24a-E-PPK | CIBT3885 | CIBT4191 |  |  | ⁕ |  | WP_000529579.1 |
| Adenylate kinase | *E. coli* | pET24a-adk | CIBT1930 | CIBT4313 |  |  |  |  | WP_123056950.1 |
| Ribonucleoside diphosphate kinase | *Saccharomyces cerevisiae* | pET24a-yes-NDK | CIBT3886 | CIBT4192 |  | ⁕ |  |  | Y Liu et al. Appl Microbiol Biotechnol 2017, 101(4): 1409-1417 |
| Ribonucleoside diphosphate kinase | *E. coli* | pET24a-eco-NDK | CIBT3887 | CIBT4193 |  |  |  | ⁕ | WP_001298109.1 |
| Ribonucleoside diphosphate kinase | *M.xanthus* | pET24a- MXAndk | CIBT3888 | CIBT4194 | ⁕ |  |  |  | WP_011553572.1 |
| Cytidylate kinase | *E. coli* | pET24a- eco-CMPK | CIBT3889 | CIBT4195 |  |  |  | ⁕ | WP_001260338.1 |
| Cytidylate kinase | *Mycobacterium tuberculosis* H37Rv | pET24a- MTcmk | CIBT3890 | CIBT4196 |  |  |  | ⁕ | Y Liu et al. Appl Microbiol Biotechnol 2017, 101(4): 1409-1417 |
| PRPP synthetase | *E. coli* | pET24a-EcPrs | CIBT1928 | CIBT4311 |  |  |  |  | WP_001119392.1 |
| PRPP synthetase | *Bacillus subtilis* | pET24a-BsPrs | CIBT1929 | CIBT4312 |  |  |  |  | WP_003218353.1 |
| Cholinephosphate cytidylyltransferase | *Saccharomyces cerevisiae* | pET28aCCT | CIBT3891 | CIBT4197 |  |  | ⁕ |  | Y Liu et al.Appl Microbiol Biotechnol 2017, 101(4): 1409-1417 |
| Cephalosporin C deacetylase | *Bacillus subtilis* 168 | pET24a-CER2 | CIBT3892 | CIBT4198 | ⁕ |  |  |  | CN101555463A |
| α-amino acid ester acyltransferase | *Sphingobacterium siyangensis* | pET24a-SsAAEA | CIBT3893 | CIBT4199 |  |  |  | ⁕ | BAK64662.1 |
| Phospholipase D | *E.coli* | pET24a-pld | CIBT3894 | CIBT4200 |  |  |  | ⁕ | WP_001259700.1 |
| Maltooligosaccharide trehalose hydrolase | *Arthrobacter* Q36 | pET24a-Q36-Mth | CIBT3895 | CIBT4201 |  |  | ⁕ |  | Karuta K et al. Biochim Biophys Acta 1996, 1289 |
| Maltooligosaccharide trehalose hydrolase | *Arthrobacter* S34 | pET24a-S34-Mth | CIBT3896 | CIBT4202 |  |  | ⁕ |  | Yamamoto T et al. Biosci Biotechnol Biochem 2001, 65(6):1419-1423 |
| Maltooligosaccharide trehalose hydrolase | *Sulfolobus acidocaldarius* | pET24a-Sa-Mth | CIBT3897 | CIBT4203 |  |  | ⁕ |  | Mukai K et al. Carbohydr Res 2005, 340 (8): 1469-74 |
| Penicillin acylase | *K.citrophila* | pET24a-KcPGA | CIBT3898 | CIBT4204 |  |  |  | ⁕ | T Cheng et al. Protein Expr Purif 2006, 46(1): 107-113 |
| Penicillin acylase | *P.rettgeri* | pET24a-pPGA | CIBT3899 | CIBT4205 |  |  |  | ⁕ |  |
| Penicillin acylase | *P.rettgeri* | pET28b-prPGA | CIBT3900 | CIBT4206 |  |  |  | ⁕ |  |
| Penicillin acylase | *Alcaligenes faecalis* | pET24a-AfPGA | CIBT3901 | CIBT4207 |  |  |  | ⁕ |  |
| Penicillin acylase | *E. coli* | pET28b-EcPGA | CIBT3902 | CIBT4208 |  |  |  | ⁕ |  |
| L-acetyl amino acid hydrolase | *Thermococcus litoralis* | pET24a-Laah | CIBT3903 | CIBT4209 |  |  |  | ⁕ | WP_004069888.1 |
| N-carbamyl-D-amino acid amidohydrolase | *Burkholderia pickettii* | pET28b-DCaseM3-E6 | CIBT3904 | CIBT4210 |  |  | ⁕ |  | D Zhang et al. Appl Microbiol Biotechnol 2011, 90(4): 1361-1371 |
| N-carbamyl-D-amino acid amidohydrolase | *Burkholderia pickettii* (A18T,Y30N,K34E ) | pET-1596DCaseM3 | CIBT3905 | CIBT4211 |  |  | ⁕ |  | S Jiang et al. Biochemical Journal 2007, 402(3): 429-437 H Yu et al. Appl Microbiol Biotechnol 2009, 82(2): 279-285 |
| N-carbamyl-D-amino acid amidohydrolase | *Burkholderia pickettii* | pET28a-Dcase | CIBT4263 | CIBT4277 |  |  |  |  |  |
| N-carbamyl-D-amino acid amidohydrolase | *Burkholderia pickettii* (A18T) | pET28a-Dcase (A18T) | CIBT4264 | CIBT4278 |  |  |  |  |  |
| N-carbamyl-D-amino acid amidohydrolase | *Burkholderia pickettii* (Y30N) | pET28a-Dcase (Y30N) | CIBT4265 | CIBT4279 |  |  |  |  |  |
| N-carbamyl-D-amino acid amidohydrolase | *Burkholderia pickettii* (K34E) | pET28a-Dcase (K34E) | CIBT4266 | CIBT4280 |  |  |  |  |  |
| D-acetylamino acid hydrolase | *Ochrobactrum anthrop* | pET24a-dap-opt | CIBT3906 | CIBT4212 | ⁕ |  |  |  | UniProtKB/Swiss-Prot: Q9ZBA9.3 |
| D-acetylamino acid hydrolase | *Alcaligenes xylosoxydans subsp*. Xylosoxydans A-6 | pET24a-NL2A | CIBT3907 | CIBT4213 |  |  | ⁕ |  | WP_026383682.1 |
| D-heinase | *Pietro restonella* CGMCC1596 | pET28b-Dhase | CIBT3908 | CIBT4214 |  | ⁕ |  |  | Z Xu et al. J Bacteriol 2003, 185(14): 4038-4049 |
| D-heinase | *Agrobacterium radiobacter* NRRL B11291 | pET28b-11291DHase | CIBT3909 | CIBT4215 |  |  |  | ⁕ | Nanba H et al. Biosci Biotechnol Biochem 1998, 62(5): 875-881 |
| Lactamase | *Comamonas acidovorans* strain CMC 4093 | pET24a-lactamase | CIBT3910 | CIBT4216 |  |  |  | ⁕ | mtmitpslhasagrtledpffptaiarstpwpetlikvdlnqspydnpqvhnrwhpdipmavwvepgaefkletydwtggaiknddsaedvrdvdlstvhflsgpvgvkgaqpgdllvvdlldigarddslwgfngffskqngggfldehfplaqksiwdfhgmftksrhipgvnfaglihpgligclpdpkmlaswneretgliatdpdripglanppnattahmgqmqgeardkaaaegartvpprehggncdikdlsrgsrvffpvyvdgaglsvgdlhfsqgdgeitfwgpiempgwvhmkvslikggmakygiknpifkpspmtpnyqglpdlrrhlggrkgqaalpgrdrglppglperhrvpeeirlqrrpgllaaghgaragphqrrggraqclrhavaahgdlrlrhqshgrgttedhhgrggsahrpgqvspaydtrhppfargrpcppmtttaphaaastrcaasrsatsprpapaarrprpasssarrawpapapnsaapttptsapgtspgahamwpraatracatpsgcgccsgaskrgstvtapngaktfptkrpwmish |
| Arginase | *Bacillus caldovelox* | pET24a-Arg | CIBT3911 | CIBT4217 |  |  | ⁕ |  | WP_014194613.1 |
| Arginase | *Bacillus brevis* TT02-8 | pET24a-BbArg | CIBT3912 | CIBT4218 |  | ⁕ |  |  | WP_012684137.1 |
| Arginine deiminase | *Lactococcus lactis ssp.* | pET28b-LADI | CIBT3913 | CIBT4219 |  |  | ⁕ |  | WP_004254504.1 |
| Arginine deiminase | *Pseudomonas putida* ATCC 4359 = NRRL B-13 | pET24a-arcA | CIBT3914 | CIBT4220 |  |  | ⁕ |  | WP_016485080.1 |
| Nitrilase | *Acidovorax facilis* 72W | pET24a-nitA | CIBT3915 | CIBT4221 |  | ⁕ |  |  | GenBank: ABD98457.1 |
| Inorganic pyrophosphatase | *Methanocaldococcus jannaschii* DSM 2661 | pET24a- Ppiase | CIBT3916 | CIBT4222 |  |  | ⁕ |  | WP_010870112.1 |
| Halohydrin dehalogenase | *Agrobacterium sp.* | pET24a-HHDH | CIBT3917 | CIBT4223 | ⁕ |  |  |  | PDB: 1PWX_A |
| Aspartic acid-α-carboxylase | *Mycobacterium tuberculosis* | pET24a-PanD-Mtb | CIBT3918 | CIBT4224 |  |  |  | ⁕ | CCE39024.1 |
| Aspartic acid-α-carboxylase | *E. coli* | pET24a-Ec-PanD | CIBT3919 | CIBT4225 |  |  |  | ⁕ | WP_000621515.1 |
| Aspartic acid-α-carboxylase | *Corynebacterium glutamicum* | pET24a-Cg-PanD | CIBT3920 | CIBT4226 |  |  |  | ⁕ | AUH99767.1 |
| L-glutamate decarboxylase | *E. coli* | pET28a-GABA | CIBT3921 | CIBT4227 |  |  | ⁕ |  | WP_000358930.1 |
| Lysine decarboxylase | *E. coli* | pET28a-cadA | CIBT1401 | CIBT4275 |  |  |  |  | Marc Lemonnier et al. Microbiology 1998, 144(3):751-760 |
| Orotate decarboxylase | *E. coli* | pET24a-pyrF | CIBT1927 | CIBT4310 |  |  |  |  | GenBank: EGI10862.1 |
| Tyrosine decarboxylase | *Lactobacillus brevis* IOEB 9809 | pET24a-tyrDC | CIBT3922 | CIBT4228 |  |  |  | ⁕ | AAN77279.2 |
| Tyrosine decarboxylase | *Methanocaldococcus jannaschii* | pET24a-mfnA | CIBT3923 | CIBT4229 |  |  | ⁕ |  | UniProtKB/Swiss-Prot: Q60358.1 |
| L-threonine aldolase | *Pseudomonas sp.* | pET24a-LTA | CIBT3924 | CIBT4230 |  |  | ⁕ |  | WP_065760197.1 |
| D-threonine aldolase | *Xanthomonas oryzae* IAM1657 | pET24a-XDTA | CIBT3925 | CIBT4231 |  |  | ⁕ |  | US 2008/0293098 A1 |
| Chloracetaldehyde aldehydase | US20110118476A1 | pET24a-DERA04 | CIBT3926 | CIBT4232 |  |  | ⁕ |  | DERA04 (US20110118476A1) |
| Chloracetaldehyde aldehydase | US20110118476A1 | pET24a-DERA102 | CIBT3927 | CIBT4233 |  |  | ⁕ |  | DERA102 (US20110118476A1) |
| Sialic aldehydase | *E. coli* | pET28-NA1 | CIBT3928 | CIBT4234 |  |  | ⁕ |  | WP_000224714.1 |
| Tryptophanase | *E. coli* | pET28a-Tryp | CIBT3929 | CIBT4235 |  |  | ⁕ |  | WP_001341776.1 |
| Tyrosine phenol-lyase | *Erwinia herbicola* MT-10509 | pET24a-TPL | CIBT3930 | CIBT4236 |  |  | ⁕ |  | AAB24234.1 |
| Nitrile hydratase | *Rhodococcus opacus* | pET24a-Nhase | CIBT3931 | CIBT4237 |  |  | ⁕ |  | NCBI: BAH96597.1; BAH96596.1 |
| Isoprene synthase | *Kudzu* (*Cyanobacteria* codon optimization) | pET28a-K1IspS | CIBT4281 | CIBT4267 |  |  |  | ⁕ | Q6EJ97.1 |
| Isoprene synthase | *Kudzu* (*E. coli* codon optimization) | pET28a-K2IspS | CIBT4282 | CIBT4268 |  |  |  | ⁕ |  |
| Isoprene synthase | *Poplulous alba* (*E.coli* codon optimization, K308R, C533W) | pET28a-P1IspS | CIBT4283 | CIBT4269 | ⁕ |  |  |  | C Yang et al. Metab Eng 2016, 37: 79-91 WO29076676A2 |
| Isoprene synthase | *Poplulous alba* (N59D, K308R, C533W) | pET28a-P2IspS | CIBT4284 | CIBT4270 |  |  |  | ⁕ | Q9AR86.1 |
| Isoprene synthase | *Poplulous alba* (human codon optimization) | pET28a-P3IspS | CIBT4285 | CIBT4271 | ⁕ |  |  |  |  |
| Isoprene synthase | *Poplulous alba* (*Cyanobacteria* codon optimization, C533W) | pET28a-P4IspS | CIBT4286 | CIBT4272 |  |  | ⁕ |  | Q9AR86.1 |
| Isoprene synthase | *Populous deltoids* | pET28a-P5IspS | CIBT4287 | CIBT4273 |  |  |  | ⁕ | AEK70966.1 |
| Isoprene synthase | *Eucalyptus* (*E. coli* codon optimization) | pET28a-e2IspS | CIBT4288 | CIBT4274 |  |  |  | ⁕ | BAF02831.1 |
| Threonine deaminase | *Bacillus subtilis* 168 | pET24a-ilvAbs | CIBT3932 | CIBT4238 |  |  | ⁕ |  | WP_003230803.1 |
| Threonine deaminase | *E. coli* W3110 | pet28b-ilvA | CIBT3933 | CIBT4239 |  |  | ⁕ |  | L Zhu et al. Appl Microbiol Biotechnol 2011, 90(3): 903-910 |
| Threonine deaminase | *E. coli* | pET24a-Tdc | CIBT3934 | CIBT4240 |  |  | ⁕ |  | WP_097728021.1 |
| Phenylalanine ammonia lyase | *R.glutinis* (*E. coli* codon optimization) | pET24a-RtPAL | CIBT3720 | CIBT4314 |  |  | ⁕ |  | XP_016272209.1 |
| Phenylalanine ammonia lyase | *Anabaena variabilis* (*E. coli* codon optimization) | pET24a-AvPAL | CIBT3721 | CIBT4315 |  |  |  | ⁕ | WP_011320679.1 |
| Alanine racemase | *B.stearothermophilus* | pET24a-BacALR | CIBT3935 | CIBT4241 |  |  |  | ⁕ | AAA22220.1 |
| Alanine racemase | *Bacillus subtilis* 168 | pET24a-dal | CIBT3936 | CIBT4242 |  |  | ⁕ |  | L Zhu et al. Appl Microbiol Biotechnol 2011, 90(3): 903-910 |
| Alanine racemase | *Achromobacter obae* | pET24a-aclr | CIBT3937 | CIBT4243 |  |  | ⁕ |  | UniProtKB/Swiss-Prot: Q7M181.1 |
| Glucosamine-2-epimerase | *Anabaena* | pET32-bage-6 | CIBT3938 | CIBT4244 |  |  | ⁕ |  | S Hu et al. Appl Microbiol Biotechnol 2011, 85(5): 1383-1391 |
| Haine racemase | *A.tumefaciens* C58 | pET24a-hr2 | CIBT3939 | CIBT4245 |  |  | ⁕ |  | WP_010972743.1 |
| Haine racemase | *Agrobacterium tumefaciens str.* | pET24a-R1 | CIBT3940 | CIBT4246 |  |  | ⁕ |  | WP_010972733.1 |
| N-acetyl amino acid racemase | *Amycolatopsis azurea* CCRC13413 | pET24a-NR3M | CIBT3941 | CIBT4247 |  |  | ⁕ |  | MKLSGVELRRVRMPLVAPFRTSFGTQSERELMLVRAVTPAGEGWGECVTMAAPVYSSEYNDAAEHVLRNHLIPALLAADDVTAYKVTPLLAKFKGHRMAKGALEMAVLDAELRAHKRSFAAELGSTRDSVACGVSVGIMDSIPQLLDVVGDYLDEGYVRIKLKIEPGWDIEPVRQVRERFGDDVLLQVDANTAYTLGDAPLLARLDPFDLLLIEQPLGEEDVHGHAELAKRIRTPICLDESIVSAKAAADAIKLGACQIVNIKPGRVGGYLEARRVHDVCAAHGVAVWCGGMIETGLGRAANVALASLPGFTLPGDTSASGRFYRTDITEPFVLEAGHLPVPTGPGLGVTPIPDILDDFTTEKVWIGS |
| N-acetyl amino acid racemase | *Alcaligenes sp.* | pET24a-AlNAR | CIBT3942 | CIBT4248 | ⁕ |  |  |  | ALX85219.1 |
| N-acetyl amino acid racemase | *Deinococcus radiodurans* NCHU1003 | pET24a-NR4 | CIBT3943 | CIBT4249 |  |  | ⁕ |  | 1R0M_A |
| N-acetyl amino acid racemase | *Amycolatopsis.sp.*TS-1-60 | pVALR | CIBT3944 | CIBT4250 |  |  | ⁕ |  | UniProt sequence Q44244 |
| Ribose-5-phosphate isomerase | *E. coli* | pET24a-ripA | CIBT1925 | CIBT4308 |  |  |  |  | A1AFA2.1 |
| Fructose isomerase | *Providencia stuartii* | pET24a-PsFI | CIBT3945 | CIBT4251 |  |  | ⁕ |  | WP_004918301.1 |
| Maltooligosaccharide trehalose synthetase | *Arthrobacter sp*.Q36 | pET24a-Q36-Mts | CIBT3946 | CIBT4252 |  |  | ⁕ |  | CN201310296116 |
| Maltooligosaccharide trehalose synthetase | *Sulfolobus acidocaldarius* | pET24a-Sa-Mts | CIBT3947 | CIBT4253 |  |  |  | ⁕ |  |
| Maltooligosaccharide trehalose synthetase | *Arthrobacter sp.* S34 | pET24a-S34-Mts | CIBT3948 | CIBT4254 |  | ⁕ |  |  |  |
| Glutamic cysteine/glutathione synthesis bifunctional enzyme | *Streptococcus agalactiae* | pET24a-SaGSH | CIBT3949 | CIBT4255 |  |  | ⁕ |  | Y Jiang et al. Appl Biochem Biotechnol 2016, 180(7): 1446-1455 |
| Glutamic cysteine/glutathione synthesis bifunctional enzyme | *Streptococcus thermophilus* | pET24a-StGSH | CIBT3950 | CIBT4256 |  |  | ⁕ |  | Y Jiang et al. Appl Biochem Biotechnol 2016, 180(7): 1446-1455 |
| Glutathione synthase 1 | *E. coli* | pET28b-gshA | CIBT3951 | CIBT4257 | ⁕ |  |  |  | WP_000611804.1 |
| Glutathione synthase 2 | *E. coli* | pET24a-gshB | CIBT3952 | CIBT4258 |  | ⁕ |  |  | WP_000593273.1 |
| CTP-synthetase | *E. coli* | pET24a-pyrG | CIBT3953 | CIBT4259 |  |  | ⁕ |  | Y Liu et al. Appl Microbiol Biotechnol 2017, 101(4): 1409-1417 |
| CTP-synthetase | *Lactococcus lactis* | pET24a-llpyrG | CIBT3954 | CIBT4260 |  |  | ⁕ |  |  |
| K+ transporter | *Saccharomyces cerevisiae* | pET21a-trk1 | CIBT4304 | CIBT4316 |  |  |  | ⁕ | MHHHHHHSSGVDLGTENLYFQSNAMHFRRTMSRVPTLASLEIRYKKSFGHKFRDFIALCGHYFAPVKKYIFPSFIAVHYFYTISLTLITSILLYPIKNTRYIDTLFLAAGAVTQGGLNTVDINNLSLYQQIVLYIVCCISTPIAVHSCLAFVRLYWFERYFDGIRDSSRRNFKMRRTKTILERELTARTMTKNRTGTQRTSYPRKQAKTDDFQEKLFSGEMVNRDEQDSVHSDQNSHDISRDSSNNNMNHNGSSVSLDDFVKEDETDDNGEYQENNSYSTVGSSSNTVADESLNQKPKPSSLRFDEPHSKQRPARVPSEKFAKRRGSRDISPADMYRSIMMLQGKHEATAEDEGPPLVIGSPADGTRYKSNVNKLKKATGINGSTIKIRDKGNESNTDQNSVSSEANSMASVSDESSLPTNFGNKVPSLRTNTHRSNSGPIAITDNGETDKKHGPSIQFDITKPPRKISKRVSTFDDLNPKSSVLYRKKASKKYLMKHFPKARRIRQQIKRRLSTGSIEKNSSNNVSDRKPITDMDDDDDDDDDNDGDNNEEYFADNESGDENERVQQSEPHSDSELKSHQQHQEKHQLQQNLHRMYKTKSFDDNRSRAVPMERSRTIDMAEAKDLNELARTPDFQKMVYQNWKAHHRKKPNFRKRGWNNKIFEHGPYASDSDRNYPDNSNTGNSILHYAESILHHDGSHKNGSEEASSDSNENIYSTNGGSDHNGLNNYPTYNDDEEGYYGLHFDTDYDLDPRHDLSKGSGKTYLSWQPTIGRNSNFLGLTRAQKDELGGVEYRAIKLLCTILVVYYVGWHIVAFVMLVPWIILKKHYSEVVRDDGVSPTWWGFWTAMSAFNDLGLTLTPNSMMSFNKAVYPLIVMIWFIIIGNTGFPILLRCIIWIMFKISPDLSQMRESLGFLLDHPRRCFTLLFPKAATWWLLLTLAGLNITDWILFIILDFDSTVVKSLSKGYRVLVGLFQSVSTRTAGFSVVDLSQLHPSIQVSYMLMMYVSVLPLAISIRRTNVYEEQSLGIYGDMGGEPEDTDTEDDGNDEDDDEENESHEGQSSQRSSSNNNNNNNRKKKKKKKTENPNEISTKSFIGAHLRKQLSFDLWFLFLGLFIICICEGDKIKDVQEPNFNIFAILFEIVSAYGTVGLSLGYPDTNQSFSRQLTTLSKLVIIAMLIRGKNRGLPYSLDRAIILPSDRLEHFDHLEGMKLKRQARTNTEDPMTEHFKRSFTDVKHRWGALKRKTTHSRNPKRSSTTL |
| **Total** |  |  |  |  | **20/196** | **27/196** | **102/196** | **47/196** |  |

**Table S4 Primers used in this study**

| **Names** | **Sequence (5’ → 3’)** |
| --- | --- |
| dns-aL-F | agaactcggtacgcgcggatcttccagagatctagaatgtgagacttacgttaaaaccg |
| dns-aL(Spc)-R | gaactcgagtagggataacagggtaataagcttagttaaagtctttaaaaagtatg |
| dns-aR(Spc)-F | gagcgtcagaccccgtagaaaagatcaaagcttcggctccatcgctcgtacctatc |
| dns-aR-R | acgccaagtttgcacgcctgccgttcgacgatctagacaatctaggggaacagtctgcc |
| Spec(dns)-F | agtcatactttttaaagactttaactaagcttattaccctgttatccctactcg |
| Spec(dns)-R | catataaagataggtacgagcgatggagccgaagctttgatcttttctacggggtctg |
| T7R(dns)-Fn | ggataaagtcatactttttaaagactttaactgcatgcccgagaagatgttgagcaaac |
| T7R(Spc)-Rn | cacatgaactcgagtagggataacagggtaatcccgggcacagtatcaaggtattttat |
| Spec(T7)-F | gtgcgcgcataaaataccttgatactgtgcccgggattaccctgttatccctactcgag |
| Spec(dns)-R | catataaagataggtacgagcgatggagccgaagctttgatcttttctacggggtctg |
